# Social signals of belonging: How the perceived ethnic‐national background of friends affects ascriptions of belonging given to descendants of migrants

**DOI:** 10.1111/bjso.12898

**Published:** 2025-05-14

**Authors:** Anniek Schlette, Tobias H. Stark, Anouk Smeekes

**Affiliations:** ^1^ Department of Interdisciplinary Social Science, ERCOMER/ICS Utrecht University Utrecht The Netherlands

**Keywords:** ascriptions, descendants of migrants, dual belonging, friends, national belonging, social categorization, vignettes

## Abstract

Many descendants of migrants feel belonging to both the national group and the ethnic minority group of their family (dual identity), but they often experience that majority members see them only as ethnic minority members. This could hamper their potential to improve intergroup relations. Because social networks tend to be homogeneous, having friends from a particular group could be interpreted as a signal of someone's group belonging. Our research advances the field by examining how the ethnic minority/national majority composition of the friendship network of descendants of migrants may affect the national/dual ascriptions they receive. In two vignette studies, we manipulated the composition of the friend group, using names or AI‐generated faces of fictitious Moroccan‐Dutch individuals, and examined how this affected ascriptions and stereotypical evaluations given by a representative sample of Dutch majority members. We find mixed results; having Dutch friends increased Dutch ascriptions and having mixed friends increased dual ascriptions when participants read text. However, these effects were attenuated when targets' faces were categorized. This suggests that information about phenotype limits the effects of friend group composition. Future research should examine these dynamics in real‐world environments where phenotype and social cues coexist.

‘I am also called “cheese.”’

“You? Based on what?”

‘I think when you hang out with Dutch people often, you are quickly called “cheese.”’

Conversation between three Moroccan‐Dutch individuals in the Dutch reality show *Poldermocro's*

In this quote, from the Dutch reality show *Poldermocro's*,^1^ a Moroccan‐Dutch individual mentions being called ‘cheese’, a joking way to refer to Dutch people, saying that he thinks he is called this way because he often hangs out with Dutch people. This quote highlights that the composition of a person's social network can be used by others to ascribe them a certain group belonging. In this paper, we examine how the ethnic minority/national majority composition of the friendship network of descendants of migrants may affect the national and dual ascriptions they receive. To the best of our knowledge, only three studies have investigated how the composition of friendship networks affects perceptions of others' group identification (Cooley et al., 2018; Johnson & Ashburn‐Nardo, 2014; Kunst et al., 2022). However, these studies were all conducted in the United States and measured perceived strength of targets' racial (self)‐identification. Hence, we advance this field by examining ascriptions of national and dual belonging given to descendants of migrants in Europe.

Drawing from self‐categorization theory (Turner, 1985), someone's friendship network may make a certain social identity more salient. As social networks are often ethnically or nationally homogeneous^2^ (Leszczensky & Stark, 2019; McPherson et al., 2001), having friends from a particular ethnic minority or national group could be interpreted by observers as a signal of this group membership. Research from the United States and Australia has shown that ethnic minority individuals who have prototypical characteristics of the national group (e.g. being overweight, liking Vegemite) were ascribed more national belonging by both minority and majority members (e.g. Handron et al., 2017; Thai et al., 2020; Yogeeswaran et al., 2012). The composition of one's friendship network might likewise be used to categorize others. If the ethnic background of friends serves as a cue of someone's social belonging, descendants of migrants with majority and minority friends might be ascribed dual belonging.

Recent research has shown that majority members who are aware of dual identifiers in their environment have more positive attitudes towards the associated outgroup (Levy et al., 2017, 2023). Recognizing dual identifiers as belonging to both the national group of the country they grew up in and the ethnic minority group of their family may thus be a key to improving intergroup relations. However, descendants of migrants often experience that majority members without a migration background see them only as ethnic minority group members (Cárdenas et al., 2021). If majority members fail to recognize others' dual identification, this potential to improve intergroup relations could be limited. Therefore, we investigate to what extent the ethnic minority/national majority background of descendants of migrants' friends affects the ascriptions of national and dual belonging given by national majority members. In addition, we examine possible downstream consequences of ascriptions on majority members' evaluation of descendants of migrants.

In two survey‐embedded vignette experiments, we manipulate the ethnic/national composition of the friend group, using names (Study 1) or AI‐generated faces (Study 2) of fictitious Moroccan‐Dutch individuals, and examine how this affects the ascriptions they receive from a representative sample of Dutch majority members.

## Ascriptions of ethnic, national and dual belonging

Social categorization is a fundamental cognitive process in which individuals classify themselves and others into social groups based on assumed shared characteristics (Tajfel, 2010). This simplifies a complex world into ingroups (‘us’) and outgroups (‘them’). Ethnic and national categories are among the most important social categories for people, and scholars agree that these categories are not fixed, but socially constructed (Barth, 1969; Brubaker, 2002; Jenkins, 1994; Verkuyten, 2018). In social categorization, two intertwined processes can be distinguished: internal and external ascription (Jenkins, 1994; Verkuyten, 2018). Internally, people self‐identify with an ethnic and/or national group, but they externally also categorize others into ethnic and/or national categories. In this paper, we focus on this external process and refer to this as *ascribing ethnic/national belonging*. Ascriptions and self‐identification do not necessarily align, and research has shown that dual identifiers often experience that one of their identities is challenged or denied by others (e.g. Cárdenas et al., 2021; Cheryan & Monin, 2005). They are, for example, asked ‘Where are you really from?’ which implies that they are not seen as part of the national group.

## The social context

Someone's social network could provide cues for categorization into certain ethnic/national groups. A key insight from social network research is that people are inclined to engage with and befriend others who are similar to them (McPherson et al., 2001). As a consequence of this homophily principle, social networks are often ethnically or nationally homogeneous (Leszczensky & Stark, 2019). Even more, people tend to overestimate and emphasize similarities between those who form a group (Tajfel & Turner, 1979). Hence, observers might use these assumed similarities to categorize groups of friends into the same ethnic/national group (Boda, 2018; Morry, 2005). In addition, self‐categorization theory (Turner, 1985) states that people have an image of what a prototypical member of a social category is, and when someone fits this prototype, they categorize them accordingly. In the United States and Australia, ethnic minority individuals who had characteristics associated with the national identity were ascribed stronger national belonging (Handron et al., 2017; Thai et al., 2020; Yogeeswaran et al., 2012). Accordingly, having national majority friends might be regarded as typical for national group members and serve as a cue for observers to categorize someone (more) as a member of the national group. In similar vein, if dual identifiers' social networks have both national majority and ethnic minority friends, this could be interpreted by observers as a social signal of someone's dual belonging.

Three experimental studies in the United States have shown that observers perceive targets' racial identity differently depending on the racial composition of targets' friend groups. Johnson and Ashburn‐Nardo (2014) showed that Black participants who viewed the Facebook page of a Black target with a White social network rated this target lower on Black identity than when the target was shown alone or with Black friends. Cooley et al. (2018) similarly found that photos of Black‐White biracial individuals who were shown with Black friends (vs alone) were rated as self‐identifying more as Black, whereas when the *same* biracial individuals were pictured with White friends (vs alone), they were seen as self‐identifying more as White. Lastly, Kunst et al. (2022) measured how the racial composition of targets' groups of friends affected people's mental image of these targets' phenotype. They found that Black targets with a darker skin tone were mentally represented as having mostly Black friends, and that Black targets with mostly Black friends were rated as less Eurocentric and more Afrocentric in their physiognomy than when these targets had equal numbers of White and Black friends or mostly White friends. Taken together, these three studies showed how targets were perceived as racially similar to their friends. Extending this work, we examine how social signals of the ethnic/national composition of friendship networks of descendants of migrants in Europe (targets) affect national and dual ascriptions given by national majority members (observers). We expect that:

- Observers give stronger *ascriptions of national belonging* to targets who only have friends with a national majority background compared to when targets only have ethnic minority friends (H1), national majority friends and ethnic minority friends (H2) or when no information is given about targets' friends (H3).
- Observers give stronger *ascriptions of dual belonging* to targets who have national majority friends and ethnic minority friends compared to when targets only have ethnic minority friends (H4), only have national majority friends (H5) or when no information is given about targets' friends (H6).

## Ethnic conceptions of nationhood

Previous research on national identity has addressed that the inclusiveness of the national category depends on someone's endorsement of an ethnic conception of nationhood (e.g. Reijerse et al., 2013; Yogeeswaran & Dasgupta, 2014). An ethnic conception of nationhood is the belief that nationhood is based on shared ancestry and descent. It is related to the principle of *jus sanguinis* (right of blood) and means that one's nationality is determined by the nationality of one's (grand)parents (e.g. Reijerse et al., 2013). This means that, for national majority members who strongly endorse an ethnic conception of nationhood, social signals of the friendship network of descendants of migrants may not affect their ascriptions of belonging. The reason is that these national majority members would exclude anyone with a migration background from national group membership (Smeekes et al., 2021; Verkuyten & Martinovic, 2015). Hence, we expect that the relationship between social signals of the friendship network and ascriptions is conditioned by the level of endorsement of an ethnic conception of nationhood. Specifically, we expect that:

- The effect of targets having only national majority friends on national ascriptions of belonging (H7) or having national majority friends and ethnic minority friends on dual ascriptions of belonging (H8) is weaker for observers who more strongly endorse an ethnic conception of nationhood.

## THE CURRENT STUDY

In this paper, we examine ascriptions given to Moroccan‐Dutch individuals by Dutch majority members. In the Netherlands, people of Moroccan descent are one of the largest ethnic minority groups and 45% of them belong to the so‐called ‘second generation’, which means that their parent(s) migrated to the Netherlands and they were born and raised in the Netherlands. The majority feel belonging to both the Moroccan and the Dutch group, making them dual identifiers (Dagevos et al., 2022). The Moroccan‐Dutch are, however, a stigmatized minority group facing negative stereotypes, such as being criminal and sexist (Gordijn et al., 2001; Grondelaers & Van Gent, 2022; Kamans et al., 2009). Moreover, a majority of Dutch people with a Moroccan background are Muslim, which is portrayed by right‐wing populist politicians as incompatible with the Christian heritage of the Netherlands (Hafez, 2014). This highlights the importance of examining national/dual ascriptions from Dutch majority members to Moroccan‐Dutch individuals.

In two vignette experiments, we manipulate the ethnic/national background of the friends of fictitious Moroccan‐Dutch individuals. We build on experimental research on ethnic/racial categorization and discrimination by using prototypical names (Study 1) and photos (Study 2) to signal the target's ethnic/national origin (e.g. Chen, 2019; Di Stasio et al., 2021; Quillian & Midtbøen, 2021). Information is signalled differently in photos compared to text. Photos are richer in information and provide visual cues that inform observers' impressions of persons (e.g. facial features, skin colour) (Harwood, 2010). Thus, considering both allows us to examine whether the social signal argument holds for different forms of social communication. We also consider the gender of targets because research on intersectionality has shown that stereotypes about ethnic minority men are more similar to stereotypes about the group, suggesting that men rather than women are considered prototypical of their ethnic minority group (Grondelaers & Van Gent, 2022; Wiemers et al., 2024). This could affect ethnic/national ascriptions, because majority members might perceive Moroccan‐Dutch men as more prototypically Moroccan than women. Therefore, in Study 2, we considered both men and women to examine to what extent the social signal argument holds across gender.

Finally, ascriptions of ethnic/national belonging can have consequences for targets because it involves in‐ and exclusion, labelling and positioning (Verkuyten, 2018). Research has, for example, found that majority students disliked peers they ascribed ethnic minority belonging to, regardless of these peers' self‐identification (Boda et al., 2020). Whether someone is ascribed ingroup belonging, outgroup belonging or both can thus affect how they are evaluated. We explore these downstream consequences by examining how our manipulation affects stereotypical evaluations of the target in terms of perceived likeability, trust and intelligence (Based on the stereotype content model, Cuddy et al., 2008). However, because we did not experimentally manipulate ascriptions, causal claims about an underlying mediation are not possible (Rohrer et al., 2022).

## STUDY 1

In Study 1, we experimentally manipulated the ethnic/national composition of the friend group of a Moroccan‐Dutch man, using names that are typical for the respective ethnic/national origins, to examine how this affects ascriptions given to him by Dutch majority members (e.g. Di Stasio et al., 2021).

### Method

#### Data and participants

Data were collected using a between‐subjects experimental design that was embedded in a larger online questionnaire about intergroup relations. The questionnaire was programmed in Qualtrics by the researchers, and the data were collected by PanelClix, a Dutch research agency. The agency provided a representative sample of Dutch majority members (i.e. both parents born in the Netherlands^3^) based on gender, age and educational level, aged 18 and older. Participants were invited to partake in the questionnaire through email and were compensated by the research agency for their participation.

An a priori power analysis showed that 612 participants were required to detect a medium effect with 90% statistical power (See Supplementary material for reasoning). In total, 863 participants completed the questionnaire. After removing those who failed the attention check (*N* = 67) or one manipulation check (*N* = 151), and removing one participant who had a missing value on the dependent variable intelligence, 644 participants remained. Of these participants, 48.76% were male, 51.09% female and 0.16% indicated ‘other’. They were on average 50.83 years old (SD = 15.56). Moreover, 15.37% had completed lower education, 51.40% medium and 33.23% higher education. Age did not differ significantly between the four manipulations (*F*(3, 640) = .74, *p* = .527, *ɳ* ^*2*^ = .003) and neither did educational level (*χ* ^*2*^(6) = 5.45, *p* = .488, Cramer's V = .065).

#### Procedure

On the first page of the questionnaire, participants read that the goal of the questionnaire was to gain insight into attitudes towards cultural groups in the Netherlands. They were informed that their participation was voluntary, and by clicking on ‘next page’, they agreed that their data would be used for academic research and that an anonymized dataset would be made publicly available. Both studies and corresponding pre‐tests were approved by the ethical committee of our university in December 2023 (FETC23‐0509), and the studies were pre‐registered at OSF (https://doi.org/10.17605/OSF.IO/T6FBR).^4^

Participants were randomly assigned to one of four manipulations of the friend group composition of a fictitious Moroccan‐Dutch man called Mohammed (See Supplementary material). Participants read a text about Mohammed saying that he is 30 years old, born and raised in the Netherlands, and that his parents were born in Morocco. He mentions that he likes to hang out with his best friends, with whom he plays soccer. Then, participants read the names of Mohammed's friends. In the *Dutch friends* condition his friends are called Tim, Jeroen, Mark and Nick. In the *Moroccan friends* condition, they are called Youssef, Hassan, Hicham and Ayoub.^5^ In the *mixed friends* (equal number of Dutch and Moroccan friends) condition, they are called Tim, Jeroen, Youssef and Hassan, thus two Dutch names and two Moroccan names. Finally, in the *no friends*/*control* condition, participants only read about Mohammed's background and that he likes to hang out with his best friends, with whom he plays soccer, but they did not see any names. A pre‐test showed that participants interpreted the names as social signals of ethnic/national origins as we intended (see Supplementary material).

After the text, participants were asked two manipulation checks. They were asked ‘In which country were the parents of Mohammed born?’ and could select one out of five countries that include the origin countries of the four largest ethnic minority groups in the Netherlands (The Netherlands, Turkey, Morocco, Suriname and Netherlands Antilles). Participants in the Dutch, Moroccan and mixed conditions were also asked ‘What was the background of Mohammed's friends?’ and could choose 1 = *All four have a Dutch background*, 2 = *Two have a Dutch background, two have a Moroccan background*, 3 = *All four have a Moroccan background and* 4 = *Other*, *namely…*.

#### Measures

*Ascriptions of national belonging* was measured by asking ‘How Dutch is Mohammed in your view?’ This measure was based on a study from Thijs and Verkuyten (2023), and is similar to those used in research on racial categorization (e.g. Cheryan & Monin, 2005). Participants could choose from a 5‐point Likert scale (1 = *not at all Dutch*, 2 = *somewhat Dutch*, 3 = *moderately Dutch*, 4 = *very Dutch* and 5 = *completely Dutch*). The variable was treated as a continuous variable, for which a higher score indicated stronger ascriptions of national belonging.

*Ascriptions of dual belonging* was created using the answers to the questions ascriptions of national belonging (NB) and ascriptions of ethnic minority belonging (EMB). Ascriptions of ethnic minority belonging was measured by asking ‘How Moroccan is Mohammed in your view?’ Participants could choose from a 5‐point Likert scale (1 = *not at all Moroccan*, 2 = *somewhat Moroccan*, 3 = *moderately Moroccan*, 4 = *very Moroccan* and 5 = *completely Moroccan*). Next, following the approach from Levy et al. (2017), we used the formula below to create a score for ascriptions of dual belonging (*DB*), resulting in a score between −1 and 5. This measure captures the relative strength of ascriptions to one group in relation to the other group; thus, it reflects a higher score when both ascription‐scores are high and there is little difference between them (See Supplementary material). Hence, it conceptually captures that dual belonging requires ascriptions to both groups to a high and similar degree (Levy et al., 2023).

$$\mathrm{DB}=\frac{\mathrm{NB}+\mathrm{EMB}}{2}-\mathrm{ABS}\left(\mathrm{NB}-\mathrm{EMB}\right)$$

*Ethnic conception of nationhood* was measured by asking participants to what extent they agreed or disagreed with two items, taken from earlier research (e.g. Verkuyten & Martinovic, 2015). Answers were measured on a 5‐point Likert scale (1 = *completely disagree*, 2 = *disagree*, 3 = *neither disagree nor agree*, 4 = *agree* and 5 = *completely agree*). The two items were ‘a real Dutchmen has Dutch ancestry’ and ‘A real Dutchmen has Dutch origins’. They had a good reliability, *r*_*SB*_ = .856 and a mean score was created. The variable was treated as continuous and mean‐centred.^6^

#### Stereotypical evaluations

Based on the stereotype content model (e.g. Cuddy et al., 2008), we assessed how the target was evaluated with three items asking: ‘how likeable/trustworthy/intelligent do you think Mohammed is?’ Participants were asked to rate the target on a 5‐point Likert scale from 1 *= not at all* to 5 = *extremely*.

#### Plan of analysis

Data handling and analyses were performed in SPSS and R. Two ANCOVA's with an interaction effect were performed. To test H1–H3, we first modelled the main effect of the experimental manipulations on *ascriptions of national belonging*. Then, a main effect of ethnic conception of nationhood was added. Finally, to test H7–H8, we added an interaction effect between the manipulation and ethnic conception of nationhood. This process was repeated to test H4–H6 but with *ascriptions of dual belonging* as the dependent variable. We tested the contrasts as specified in our hypotheses (See Supplementary material). Exploratively, we also examined the contrasts for which we had no prior expectations, using Tukey's HSD for multiple comparisons. Then, we explored the associations between the manipulations and the stereotypical evaluations, and the ascriptions and the stereotypical evaluations. Finally, to check the robustness of our results, we repeated our analyses including those who failed the attention check or any manipulation check because those who failed these checks might not be a random sub‐sample (e.g. Kotzian et al., 2020).

### Results

#### Descriptive results

Table 1 shows the descriptive results for the ascription variables and ethnic conception of nationhood per friend group condition. As expected, participants gave the strongest Dutch ascriptions in the Dutch friends condition and the lowest in the Moroccan friends condition. Moreover, the average scores of dual ascriptions were quite low as the scale ranged from −1 to 5.

**TABLE 1:** Means and standard deviations of the ascription variables and ethnic conception of nationhood in the four conditions (*N =* 644).

	Friend group conditions				Test of mean differences (ANOVA)		
	Dutch friends (*N* = 168)	Moroccan friends (*N* = 144)	Mixed friends (*N* = 148)	No friends (control) (*N* = 184)			
	*M* (SD)	*M* (SD)	*M* (SD)	*M* (SD)	*F*	*p*	*ɳ* ^2^
Dutch ascription	3.64_a_ (1.07)	2.53_b_ (1.04)	3.45_ac_ (1.06)	3.34_c_ (1.06)	32.04	<.001	.131
Dual ascription	1.22_a_ (1.16)	1.40_ab_ (1.28)	1.65_b_ (1.38)	1.42_ab_ (1.18)	3.07	.027	.014
Moroccan ascription	2.36_a_ (.91)	3.09_b_ (1.04)	2.64_a_ (1.00)	2.58_a_ (.97)	14.97	<.001	.066
Ethnic conception of nationhood	3.11_ab_ (1.12)	3.15_ab_ (1.09)	2.99_a_ (1.06)	3.37_b_ (1.13)	3.64	.013	.017

*Note*: Standard deviations are presented in parentheses; means with different subscripts differed at *p* < .05 in Tukey's HSD tests.

#### Ascriptions of belonging

We expected that Dutch ascriptions would be higher in the Dutch friends condition compared to the Moroccan friends (H1), mixed friends (H2) or no friends condition (H3). As shown in Table 1, there was a significant effect for the friends condition on Dutch ascriptions, with a large effect size. A Tukey's HSD test showed that the target received significantly higher Dutch ascriptions in the Dutch friends condition compared to the Moroccan friends (*Mean diff =* 1.12, *p adj* < .001) and no friends condition (*Mean diff =* .30, *p adj* = .041). However, there was no difference in Dutch ascriptions between the Dutch friends condition and mixed friends condition (*Mean diff =* .20, *p adj* = .353). Thus, we found support for H1 and H3, but not for H2.

We expected that dual ascriptions would be higher in the mixed friends condition compared to the Moroccan friends (H4), Dutch friends (H5) or no friends condition (H6). Table 1 shows a significant effect for the friends condition on dual ascriptions. A post‐hoc Tukey's HSD test showed that the target was only ascribed significantly more dual belonging in the mixed friends compared to the Dutch friends condition (*Mean diff =* .43, *p adj* = .014). Although dual ascriptions were higher in the mixed condition compared to the Moroccan friends and control condition, these mean differences were not significant. This supports H5, but not H4 or H6.

Additionally, we expected that the positive effect for the Dutch friend condition on Dutch ascriptions (H7) and the effect of the mixed friend condition on dual ascriptions (H8) would be weaker for high ethnic nationhood endorsers. For Dutch ascriptions, there was no significant interaction effect of friends condition by ethnic conception of nationhood (*F*(3, 636) = .46 *p* = .709, *ɳ*_*p*_^*2*^ = .002), and thus no support for H7. Instead, stronger endorsement of ethnic nationhood was associated with lower ascriptions of Dutch belonging regardless of the friend group (see Supplementary material). For dual ascriptions, there was a significant interaction (*F*(3, 636) = 6.99, *p* < .001, *ɳ*_*p*_^*2*^ = .032), but there were no significant differences between the mixed friends condition and any other friend group conditions for high ethnic nationhood endorsers. Thus, no support for H8 was found. There were some additional findings, which are discussed in the Supplementary material.

#### Stereotypical evaluations

A MANCOVA analysis for likeability, trust and intelligence showed that there were main effects of the friends condition (*F*(9, 1908) = 7.99, *p* < .001) and ethnic conception of nationhood (*F*(3, 634) = 45.81, *p* < .001), but there was no interaction effect (*F*(9, 1908) = .47, *p* = .895). To further examine the main effect of the friend group condition, separate ANCOVAs were conducted for all three dependent variables. A post‐hoc Tukey HSD showed that the target was rated significantly more likeable and intelligent in the Dutch or mixed friends condition compared to the Moroccan or no friends condition, and more trustworthy in the Dutch friends condition compared to the Moroccan friends or no friends condition (see Supplementary material).

#### Robustness check and additional analyses

As a robustness check, we repeated the analyses including participants who failed the attention or manipulation check (*N* = 860). For Dutch ascriptions, the ANOVA results were similar, but the post‐hoc test revealed that there was no significant difference between the Dutch friends and no friends condition (*Mean diff =* −.025, *p adj* = .104). For dual ascriptions, there was no main effect of the friends condition (*F*(3, 856) = 1.54, *p* = .203, *ɳ* ^*2*^ = .005), but there was still a similar interaction effect (*F*(3, 852) = 5.16, *p* = .002, *ɳ*_*p*_^*2*^ = .018). For the stereotypical evaluations, the results were similar (see Supplementary material).

For additional analyses, participants were asked how close they thought the friend group was and to what extent they thought the target could be a bridge between the Dutch and Moroccan community in the Netherlands. There were no significant differences in closeness between the friends conditions, but the target was perceived to have less bridging potential in the Moroccan friends condition compared to the Dutch, mixed, and no friends condition (see Supplementary material).

### Conclusion

Overall, we found that participants ascribed more national belonging to the target in the national majority friends condition compared to the ethnic minority friends or no friends (control) condition. Moreover, participants ascribed more dual belonging to the target in the mixed friends condition compared to the national majority friends condition. The target was also rated more likeable and intelligent in the national majority and mixed friends conditions compared to the ethnic minority or no friends conditions, and more trustworthy in the national majority friends condition compared to the ethnic minority or no friends condition. Finally, stronger endorsement of ethnic nationhood was associated with lower ascriptions of national belonging in all friend group conditions, and the effect of the mixed friends condition on dual ascriptions was not weaker for high ethnic nationhood endorsers.

## STUDY 2

In the second study, we extended Study 1 in two ways. First, we experimentally manipulated the ethnic/national composition of the friend group of Moroccan‐Dutch targets using AI‐generated faces that looked either prototypically Dutch or Moroccan; and, second, we considered both male and female targets.

### Method

#### Data and participants

Similar to Study 1, data were collected with a between‐subjects experiment embedded in an online questionnaire, and the same research agency provided a new representative sample of Dutch majority members. The study and corresponding pre‐tests were approved by the ethical committee of our university in December 2023 (FETC23‐0509) and pre‐registered at OSF (https://doi.org/10.17605/OSF.IO/T6FBR).^7^ An a priori power analysis showed that 665 participants were required to detect a medium effect with 90% statistical power (see Supplementary material for reasoning). In total, 940 participants completed the questionnaire. After removing those who failed the attention check (*N* = 73) or had a missing value on a dependent variable (*N* = 6), 861 participants remained (see Supplementary material S1 for a sample description).

#### Procedure

In our experiment, we used three male and three female Moroccan‐Dutch targets. Each target was presented in every friend group condition. Hence, we used 14 AI‐generated faces from Generated Photos,^8^ of four prototypical Dutch men and women and 10 prototypical Moroccan men and women. These faces were selected based on the results of a pre‐test in which a representative online sample of 1477 Dutch majority members rated 194 AI‐generated faces on: realness, attractiveness and how prototypical Dutch and Moroccan looking they were (see Supplementary material). Our targets were the six faces that were rated as most prototypical Moroccan‐looking (three men, three women) in the pre‐test.

Participants were randomly assigned to one out of 24 conditions in a 2 (male or female target) × 4 (Dutch friends, Moroccan friends, mixed friends, no friends) × 3 (target 1, target 2 or target 3) between‐subjects design. Targets only had friends of the same gender because friendships between men and women are less common (McPherson et al., 2001). Participants read a similar introduction as in Study 1, stating that they were shown a fictitious^9^ person (the target), who is 30 years old, was born and raised in the Netherlands and has parents who were born in Morocco. Then, they saw a picture of this person with his/her two best friends and were told that they like to hang out and that the friends are very important to the target. In the *Dutch friends* condition, the target (one of the Moroccan‐looking faces) was presented with two Dutch‐looking faces (the friends). In the *Moroccan friends* condition, the target was shown with two Moroccan‐looking faces. In the *mixed friends* condition, the target was presented with one Moroccan‐looking face and one Dutch‐looking face. In the *no friends* condition, participants saw the target alone. A red circle indicated the target (see Supplementary material).

#### Measures

We used the same measures as in Study 1 for *ascription of national belonging*, a*scription of dual belonging*, *ethnic conception of nationhood* (*r*_*SB*_ = .864) and *stereotypical evaluations*. In addition, we asked ‘how attractive do you find this person?’ because this can impact overall impressions.

#### Plan of analysis

Data handling and analyses were performed in SPSS and R. We had the same expectations as in Study 1, and compared the same contrasts. To check if the three male/female targets differed, two two‐way ANOVAs for the manipulation and the three targets on all dependent variables were performed (see Supplementary material). This showed that there was only a difference between the three male/female targets for likeability, and an interaction effect for Dutch ascriptions for female targets between the manipulation and the targets. Although this indicated that the three female/male targets were rated differently on likeability and Dutch ascriptions, repeating the main analyses excluding the deviating target showed that the conclusions remained the same (see Supplementary material). Accordingly, we proceeded with the pre‐registered 4 (friends) × 2 (gender) design for the main analyses.

To test our hypotheses, we conducted separate ANCOVA's with interaction effects for the dependent variables. In these models, we first estimated a main effect for the friend group. Second, we added ethnic conception of nationhood, and then, we added the interaction effect between the friend group and ethnic conception of nationhood. In addition, we explored to what extent the social signal argument worked differently across male and female targets. For these multiple comparisons, we corrected with Tukey's HSD. Then, we explored the associations between the friend group and the stereotypical evaluations, and the ascriptions and the stereotypical evaluations. Finally, we conducted a robustness check of our results by repeating the analyses including participants who failed the attention check.

### Results

#### Descriptive results

Similar to Study 1, participants gave the strongest Dutch ascriptions in the Dutch friends condition (Table 2). The average scores of dual ascriptions were again comparably low because the scale ranged from −1 to 5. On average, the male targets (*M =* 2.55, *SD =* 1.05) and female targets (*M =* 2.60, *SD =* 1.04) were ascribed almost equal levels of Dutch belonging (*t*(858.73) = −.79, *p =* .430).

**TABLE 2:** Means and standard deviation of the ascription variables and ethnic conception of nationhood by friend group condition (*N* = 861).

	Friend group conditions							
	Men				Women			
	Dutch friends (*N* = 111)	Moroccan friends (*N* = 108)	Mixed friends (*N* = 105)	No friends (control) (*N* = 105)	Dutch friends (*N* = 106)	Moroccan friends (*N* = 107)	Mixed friends (*N* = 110)	No friends (control) (*N* = 109)
	M (SD)	M (SD)	M (SD)	M (SD)	M (SD)	M (SD)	M (SD)	M (SD)
Dutch ascriptions	2.71_a_ (1.07)	2.45_a_ (1.04)	2.50_a_ (1.04)	2.52_a_ (1.05)	2.81_a_ (1.08)	2.58_ab_ (1.10)	2.62_ab_ (1.00)	2.41_b_ (.95)
Moroccan ascriptions	2.66_a_ (.95)	2.69_a_ (1.00)	2.63_a_ (.94)	2.90_a_ (.96)	2.46_a_ (.95)	2.84_b_ (.93)	2.54_a_ (1.00)	2.90_b_ (.97)
Dual ascriptions	1.37_a_ (1.04)	1.26_a_ (1.06)	1.32_a_ (1.00)	1.40_a_ (1.15)	1.25_a_ (1.02)	1.44_a_ (1.18)	1.26_a_ (1.06)	1.51_a_ (1.22)
Ethnic conception of nationhood	3.24_a_ (1.07)	3.17_a_ (1.07)	3.22_a_ (1.11)	3.34_a_ (1.14)	3.17_ab_ (1.07)	3.21_ab_ (1.12)	3.04_a_ (1.12)	3.46_b_ (1.03)

*Note*: Within men and women, means with different subscripts differed at *p* < .05 in Tukey's HSD tests.

#### The effect of friends

For male targets, the friend group condition did not affect Dutch ascriptions (*F*(3, 425) = 1.29, *p =* .278, *ɳ* ^*2*^ = .009) or dual ascriptions (*F*(3, 425) = .37, *p =* .773, *ɳ* ^*2*^ = .003). For the female targets, there was a main effect of the friend group condition on Dutch ascriptions (*F*(3, 428) = 2.68, *p =* .046, *ɳ* ^*2*^ = .018). The only significant contrast was that in the Dutch friends condition the female targets were ascribed more Dutch belonging compared to the no friends condition (*Mean diff* = .40, *p adj* = .026). For female targets, the friend group condition did not affect dual ascriptions (*F*(3, 428) = 1.45, *p =* .228, *ɳ* ^*2*^ = .010). Therefore, no support was found for H1, H2, H4, H5 or H6, and H3 was only supported for female targets. In addition, the friend group condition did not affect likeability, trust or intelligence for male or female targets, and only affected attractiveness for male targets (*F*(3, 425) = 2.85, *p =* .037, *ɳ* ^*2*^ = .020). A post‐hoc test showed that in the mixed friends condition male targets were seen as less attractive than in the Moroccan friends condition (*Mean diff* = −.37, *p adj* = .029). See Supplementary material for all ANOVA results.

#### Targets' gender

To examine the effect of gender, ANCOVA's with the friend group condition, targets' gender and an interaction were performed for all dependent variables. These analyses showed no significant interaction effects, which means that the effect of the friend group condition did not differ for male and female targets (see Supplementary material).

#### Ethnic conception of nationhood

To test H7 and H8, ANCOVAs for the ascription variables were performed with the friend group condition, ethnic conception of nationhood and their interaction (see Supplementary material). For the male targets, these analyses showed no significant interaction effect on any of the dependent variables. Rather, stronger endorsement of ethnic nationhood was associated with lower ascriptions of Dutch and dual belonging and lower stereotypical evaluations in all friend group conditions.

For female targets, there was a significant interaction effect for Dutch ascriptions (*F*(3, 424) = 2.97, *p =* .032, *ɳ*_*p*_^*2*^ = .021), but not for dual ascriptions (*F*(3, 424) = .37, *p =* .772, *ɳ*_*p*_^*2*^ = .003). Moreover, post‐hoc Tukey HSD's of separate ANOVA's showed that high ethnic nationhood endorsers gave less Dutch ascriptions in the mixed friends compared to the Dutch friends condition (*Mean diff* = −.87, *p adj* = .043), but no other contrasts were significant (see Supplementary material). Thus, no support for H7 or H8 was found.

#### Robustness check and additional analyses

As a robustness check, all analyses in Study 2 were repeated including participants who had failed the attention check (*N* = 933) and the conclusions remained the same, but some additional results were found (see Supplementary material). Similar to Study 1, we also examined the closeness of the friend group and the bridging potential of the target. Neither closeness nor bridging potential of male or female targets differed between friend group conditions or based on targets' gender (see Supplementary material).

### Conclusion

Overall, we found different results for male targets compared to female targets for national ascriptions. For male targets, there was no effect of the friend group on national ascriptions, while female targets were ascribed higher levels of national belonging in the national majority friend condition compared to the no friends (control) condition. For neither male nor female targets was there an effect of friend group on dual ascriptions or stereotypical evaluation. Finally, stronger ethnic nationhood endorsement was associated with less national and dual ascriptions as well as lower stereotypical evaluations for male targets regardless of the friend group condition. Additionally, higher ethnic nationhood endorsers gave less national ascriptions to female targets in the mixed friends condition compared to the national majority friends condition.

## GENERAL DISCUSSION

Across two vignette experiments, we examined how social signals of the ethnic minority/national majority composition of descendants of migrants' friend groups affected ascriptions of national and dual belonging and stereotypical evaluations given by national majority members. Specifically, using prototypical names (Study 1) and AI‐generated faces (Study 2), we manipulated whether descendants of migrants socially signalled that they had national majority, ethnic minority or mixed friends, or that no information about friends was provided.

First, we expected that targets would be ascribed more national belonging when they had national majority friends (H1–H3). In Study 1, the (male) target with national majority friends was ascribed significantly more national belonging compared to when he had ethnic minority, mixed or no friends. Moreover, he was also seen as more likeable, trustworthy and intelligent. However, these effects were not replicated for male targets in Study 2. In this study, female targets with national majority friends were ascribed more national belonging than those with no friends. The findings of Study 1 and for female targets in Study 2 are in line with self‐categorization theory (Turner, 1985) and racial identity perception research (e.g. Cooley et al., 2018), suggesting that observers can perceive people's national group belonging differently depending on the number of national majority friends in their friendship network.

Regarding dual ascriptions, we expected that targets would be ascribed more dual belonging when they had mixed friends (H4–H6). In Study 1, mixed friends increased dual ascriptions compared to national majority friends. Similar to when the target had national majority friends, he was perceived as part of the national group, but with mixed friends he was also seen as an ethnic minority member. In other words, a diverse social network enhanced recognition of a dual identity. Moreover, the target with mixed friends received comparable ratings of likeability and intelligence to when he had national majority friends. Hence, a few social connections with national majority individuals (i.e. two national majority friends, half of the friend group) facilitated positive evaluations by national majority members. These findings suggest that descendants of migrants who engage with both their parents' heritage culture and the dominant society (i.e. integration; Berry, 1997) are positively evaluated, and their dual identity is more easily recognized by majority members.

Nonetheless, our findings only partially support the idea that perceived social signals of the social network affect national and dual ascriptions because our findings from Study 1 were not replicated in Study 2. In Study 2, the friend group neither significantly affected national ascriptions for male targets nor did it affect dual ascriptions or stereotypical evaluations for male or female targets. First, a possible explanation for the absence of an effect for male targets (while we found an effect for female targets) is the prototypicality of the male national majority friends in the AI‐generated faces. Dutch people are prototypically imagined with blond hair. The pictures of the female Dutch friends were generated with blond hair, but the male Dutch friends were not. As a result, the male Dutch friends stimuli may not have been recognized as being Dutch by participants, which reduced their impact on national ascriptions. Secondly, the absence of a friend group effect in Study 2 may reflect differences in participants' responses to textual versus visual stimuli. Unlike text (Study 1), AI‐generated faces (Study 2) provided rich visual cues, such as skin tone or hairstyle, that can affect judgement (e.g. Feliciano, 2016). Perhaps, when presented with a face, people primarily rely on phenotype to ascribe ethnic/national belonging to others and less on someone's friends.

Finally, we expected that participants who more strongly endorsed an ethnic conception of nationhood would give lower national ascriptions when the target had national majority friends, and lower dual ascriptions when the target had mixed friends (H7–H8). However, there was no evidence for these hypotheses in Study 1 or Study 2. Hence, contrary to our theoretical reasoning (e.g. Smeekes et al., 2021; Verkuyten & Martinovic, 2015), the perceived social signals of having national majority or mixed friends had the same effect on ascriptions regardless of national majority members' strength of ethnic nationhood beliefs. Nonetheless, a stronger endorsement of an ethnic conception of nationhood was negatively associated with national ascriptions and positive evaluations for male targets in both studies. Moreover, high ethnic nationhood endorsers gave more national ascriptions to female targets with only national majority friends compared to mixed friends. This suggests that for high ethnic nationhood endorsers having one national majority friend is ‘not enough’ to be considered part of the national group.

### Limitations and future research

This study has several limitations. First, we only examined prototypical names and faces of Moroccan‐Dutch individuals as cues for social signalling the friend group composition. Therefore, the generalizability of our results is limited to prototypical Moroccan‐Dutch individuals. Future research should extend our work to targets and friends with less prototypical names and varying phenotypes. Second, there are likely other factors of the target, like age, social‐economic status or religiosity, which were not varied in this study but can affect ascriptions and stereotypical evaluations (e.g. Freeman et al., 2011). Future research could also explore if our findings generalize to cross‐gender friendships.

Third, Study 2 lacked a manipulation check, leaving uncertainty about whether participants recognized the ethnic composition of the friend group as intended. It thus remains unclear whether the null findings are due to a failed manipulation or because the visual stimuli shifted participants' attention from the composition of the friend group to the phenotype of the targets. Future research could disentangle these effects by providing faces for the friend group and experimentally manipulating whether a face is shown for the target person or not. Lastly, the target had only two friends in Study 2, while the target in Study 1 had four friends. This difference could have also caused the different findings because more friends might have provided a stronger manipulation. Future studies could explore to what extent the number of friends or different ethnic/national compositions matters for person perceptions.

## CONCLUSION

To conclude, our findings show that the ethnic‐national composition of friendship networks can shape ascriptions and stereotypical evaluations given to descendants of migrants by national majority members. As expected, having national majority friends increased ascriptions of national belonging and positive evaluations for a Moroccan‐Dutch man described in the text and for AI‐generated faces of Moroccan‐Dutch women. Moreover, an ethnically diverse social network increased dual ascriptions and positive evaluations for a Moroccan‐Dutch man described in the text. However, when AI‐generated faces were shown, the friend group composition neither affected ascriptions for Moroccan‐Dutch men nor stereotypical evaluations for men or women. Future research should investigate how social signals operate in real‐world settings that provide information about someone's phenotype but also contextual information about people's social networks to better understand the interplay between physical appearance and social network characteristics in shaping national and dual identity ascriptions.

## AUTHOR CONTRIBUTIONS

**Anniek Schlette:** Conceptualization; writing – original draft; writing – review and editing; formal analysis; methodology. **Tobias H. Stark:** Conceptualization; funding acquisition; writing – review and editing; methodology; supervision. **Anouk Smeekes:** Conceptualization; methodology; writing – review and editing; supervision.

## FUNDING INFORMATION

This study was funded by the European Union (ERC, DUALNETS, 101043732). Views and opinions expressed are, however, those of the author(s) only and do not necessarily reflect those of the European Union or the European Research Council Executive Agency. Neither the European Union nor the granting authority can be held responsible for them.

## CONFLICT OF INTEREST STATEMENT

The authors declare that there is no conflict of interest.

## Supporting information


Data S1.

## Data Availability

The data and code that support the findings of this study are openly available in OSF at https://osf.io/j25wu/. DOI 10.17605/OSF.IO/J25WU.

## Footnotes

^1^
Poldermocro's was a Dutch reality show produced by BNNVARA which followed six Moroccan‐Dutch individuals who go to Morocco, the birth country of their parents, to uncover their roots and identity. https://npo.nl/npo3/poldermocro‐s/BV_101408392.

^2^
We use ‘ethnic’ in Weber's (1968) sense of the belief in common descent and ancestry. In the European context, the label for the ethnic majority group is typically the same as for the national group. Descendants of migrants can thus not be ethnically Dutch while still being considered Dutch nationals.

^3^
This is in line with the definition of people without a migration background by Statistics Netherlands (CBS, 2024). By definition, those who have grandparents who were born outside of the Netherlands (third generation) could be part of our sample; however, this possibility is small because third generation non‐western migrants in the Netherlands are a small group who are still young (majority below 18 years) (CBS, 2016).

^4^
A first version of the introduction and methods of this paper was accepted as a registered report. The few minor changes of the final manuscript compared to the Stage 1 submission or the pre‐registration are described in the Supplementary material.

^5^
These names were chosen because they were the most popular names given by Dutch parents or the most common names in the Netherlands given by Moroccan parents in the beginning of the 1990s (Nederlandse Voornamenbank, 2024).

^6^
We deviated from our pre‐registration because we incorrectly stated that a third item would be used to measure ethnic conception of nationhood, namely ‘a real Dutchmen is attached to the traditional Dutch lifestyle’. However, this item theoretically captures a cultural conception of nationhood (Reijerse et al., 2013). As a robustness check, we conducted the analyses including the third item, which did not change the main conclusions.

^7^
The few changes of the final manuscript compared to the Stage 1 submission or the pre‐registration are described in the Supplementary material.

^8^
Generated Photos (https://generated.photos/datasets).

^9^
In a pilot study, we tested whether using the word ‘fictitious’ compared to not mentioning this in our experiment affected Dutch ascriptions and likeability. We did not find a difference for male nor female targets.
