# Supplementary material for: Social signals of belonging: How the perceived ethnic‐national background of friends affects ascriptions of belonging given to descendants of migrants
Source: Br J Soc Psychol. 2025 May 14;64(3):e12898. doi: 10.1111/bjso.12898 (PMC12079013; doi:10.1111/bjso.12898)
Supplement: Supplementary file 1 — Data S1. [file BJSO-64-0-s001.docx]

**Social Signals of Belonging: How the Perceived Ethnic-National Background of Friends Affects Ascriptions of Belonging Given to Descendants of Migrants**

**Supplementary Material**

Table of Content

[Study 1: Pre-test 2](#_Toc196306457)

[Study 1: Vignettes 3](#_Toc196306458)

[Ascriptions of Dual Belonging 4](#_Toc196306459)

[Planned Contrasts ANCOVA’s 5](#_Toc196306460)

[Sample Size Reasoning 6](#_Toc196306461)

[Study 2: Pre-test AI-generated Photos 7](#_Toc196306462)

[Study 2: Results Pre-test AI-generated Photos 9](#_Toc196306463)

[Study 2: Vignettes Friend Group Conditions 14](#_Toc196306464)

[Study 1: Ethnic Conception of Nationhood 16](#_Toc196306465)

[Study 1: ANCOVA Results of Evaluations 18](#_Toc196306466)

[Study 1: Results Robustness Check 19](#_Toc196306467)

[Study 1: Additional Analysis 21](#_Toc196306468)

[Study 2: Target Differences 22](#_Toc196306469)

[Study 2: Robustness Without Target 1 26](#_Toc196306470)

[Study 2: Friend Group Conditions 28](#_Toc196306471)

[Study 2: Descriptive Results Evaluations 29](#_Toc196306472)

[Study 2: Effect of Targets’ Gender 30](#_Toc196306473)

[Study 2: Sample Description 31](#_Toc196306474)

[Study 2: Friend Condition and Ethnic Nationhood 32](#_Toc196306475)

[Study 2: Results Robustness Checks 36](#_Toc196306476)

[Study 2: Additional Analyses 40](#_Toc196306477)

[Deviation of Final Study to Stage 1 or OSF Preregistration 41](#_Toc196306478)

# Study 1: Pre-test

In Study 1, we used names to signal the respective ethnic/national origins of target’s friends. In a pre-test, we examined if participants correctly interpreted these names as signals of a certain ethnic/national origin. Therefore, we tested two versions of the text: only names of friends, and names with explicitly mentioning the ethnic/national background of friends. In an online questionnaire, a representative sample of 589 Dutch majority members were randomly assigned to one of six conditions (2: text version x 3: Dutch friends, Moroccan friends, or mixed friends). Participants only answered two friend condition checks: “In which country were the parents of Mohammed born?”, answer options were The Netherlands, Turkey, Morocco, Suriname, Netherlands Antilles, and “What was the background of Mohammed’s friends?” (1 = *All four have a Dutch background,* 2 = *Two have a Dutch background, two have a Moroccan background,* 3 = *All four have a Moroccan background,* 4 = *Other, namely…*). We were interested to see if participants answered the second friend condition check differently across the two text versions because that would indicate that explicitly mentioning ethnic/national background resulted in different answers.

Using cross-tabulations, we compared the answers given to the question on the background of Mohammed’s friends for the three conditions across the two versions. Three chi-square tests showed that there was no significant difference in answers given between the two versions in the Dutch condition (χ^2^(3) = 4.107, *p* = .250), the Moroccan condition (χ^2^(3) = 2.240, *p* = .524), or the mixed condition (χ^2^(3) = 2.938, *p* = .401). This indicates that the ethnic/national signals of the names were strong and interpreted by participants as intended. Hence, we will use the text version in which we only use the names of friends to signal ethnic/national background.

# Study 1: Vignettes

In Study 1, ethnic/national background of friends was signalled using names. The introduction and vignettes that were presented to participants were held constant across conditions. Below are the vignettes as translated from Dutch, with the friend condition underlined.

*Introduction*

On the next page, you will read a description of a fictitious person named Mohammed. We are interesting in your impression of him and will ask you questions about him. Please read the text carefully, you cannot return to the text if you go to the next page.

*Condition 1 (Dutch friends)*

My name is Mohammed and I am 30 years old. I was born and raised in the Netherlands and my parents were born in Morocco. In my leisure time I like to hang out with my best friends. Their names are Tim, Jeroen, Mark and Nick. We met each other at a soccer club and like to play soccer together. My friends are very important to me.

*Condition 2 (Moroccan friends)*

My name is Mohammed and I am 30 years old. I was born and raised in the Netherlands and my parents were born in Morocco. In my leisure time I like to hang out with my best friends. Their names are Youssef, Hassan, Hicham and Ayoub. We met each other at a soccer club and like to play soccer together. My friends are very important to me.

*Condition 3 (mixed friends)*

My name is Mohammed and I am 30 years old. I was born and raised in the Netherlands and my parents were born in Morocco. In my leisure time I like to hang out with my best friends. Their names are Tim, Jeroen, Youssef and Hassan. We met each other at a soccer club and like to play soccer together. My friends are very important to me.

*Condition 4 (no friends/control)*

My name is Mohammed and I am 30 years old. I was born and raised in the Netherlands and my parents were born in Morocco. In my leisure time I like to hang out with my best friends. We met each other at a soccer club and like to play soccer together. My friends are very important to me.

# Ascriptions of Dual Belonging

Following Levy et al. (2017), we created ascriptions of dual belonging (DB) by combining the scores of ascriptions of national belonging (NB) and ascriptions of ethnic minority belonging (EMB). The proposed formula, mathematically identical to the formula for attitude ambivalence created by Thompson et al. (1995), takes into account the relative strength of both ascriptions. As shown in Table S1, this allows for measuring both the strength of both ascriptions as well as the difference between them. A higher score thus reflects that both national and ethnic minority ascriptions were simultaneously strongly ascribed.

$$DB=\frac{NB+EMB}{2}-ABS(NB-EMB)$$

*Table S1. Possible scores for ascriptions of dual belonging.*

|  |  | Dutch belonging | | | | |
| --- | --- | --- | --- | --- | --- | --- |
|  |  | **1** | **2** | **3** | **4** | **5** |
| Ethnic minority belonging | **1** | 1 | 0.5 | 0 | -0.5 | -1 |
|  | **2** | 0.5 | 2 | 1.5 | 1 | 0.5 |
|  | **3** | 0 | 1.5 | 3 | 2.5 | 2 |
|  | **4** | -0.5 | 1 | 2.5 | 4 | 3.5 |
|  | **5** | -1 | 0.5 | 2 | 3.5 | 5 |

# Planned Contrasts ANCOVA’s

Table S2 shows the contrast between the friend conditions which were modelled to test the corresponding hypotheses.

*Table S2. Planned contrasts.*

|  | **Independent variable: contrasts** | **Dependent variable** |
| --- | --- | --- |
| H1 | Ethnic minority vs. National majority | Ascriptions of national belonging |
| H2 | Mixed vs. National majority | Ascriptions of national belonging |
| H3 | Control vs. National majority | Ascriptions of national belonging |
| H4 | Ethnic minority vs. Mixed | Ascriptions of dual belonging |
| H5 | National majority vs. Mixed | Ascriptions of dual belonging |
| H6 | Control vs. Mixed | Ascriptions of dual belonging |
|  |  |  |
| Explorative | Control vs. Ethnic minority | Ascriptions of national belonging |
| Explorative | Mixed vs. Ethnic minority | Ascriptions of national belonging |
| Explorative | Control vs. Mixed | Ascriptions of national belonging |
| Explorative | Control vs. Ethnic minority | Ascriptions of dual belonging |
| Explorative | National majority vs. Ethnic minority | Ascriptions of dual belonging |
| Explorative | Control vs. National majority | Ascriptions of dual belonging |

# Sample Size Reasoning

**Study 1**

To determine our sample size, we performed an a-priori power analysis using the InteractionPoweR Shiny app, for interactions in linear regression (Finsaas et al., 2021). Similar research (Cooley et al., 2018) has found medium effect sizes, therefore we selected an effect size of Cohen’s *f* of .25 for our main relations, and a small effect of .12 for the interaction. For an experiment with four conditions, the power analysis revealed that a total sample of 612 would be required to ensure 90% statistical power with an alpha of .05. We aimed for a total sample size of 862, with 215 participants in each condition^[[1]](#footnote-1)^, to account for excluding participants who: (1) did not finish the questionnaire because they were given the right to stop at any time, (2) failed the attention check at the beginning of the questionnaire ( ‘Please select agree’ on a five-point scale (1 *= completely disagree*, 5 *= completely agree*) and (3) who failed one or both of the manipulation checks that ensured that participants understood the experimental prompt.

**Study 2**

Similar to Study 1, we performed an a-priori power analysis using the InteractionPoweR Shiny app (Finsaas et al., 2021). For an experiment with 24 conditions, 2 (gender target) x 4 (friendship composition) x 3 targets (faces), the analysis revealed that a total sample of 665 would be required to ensure 90% statistical power to detect an effect size of Cohen’s *f* = .25 for the main-effects and *f* = .12 for the interaction-effect, given an alpha of .05. Our inclusion criteria were similar to Study 1, thus, we aimed for a larger sample of 937, with 39 participants per condition.

# Study 2: Pre-test AI-generated Photos

In Study 2, we signalled ethnic/national origin with AI-generated faces that looked either prototypically Dutch or Moroccan. We opted for creating AI-generated faces and pre-testing these because the databases we were aware of had no Moroccan-Dutch women (Langner et al., 2010), or no neutral facial expressions (van der Schalk et al., 2011) or had shown that majority members did not consistently recognise Arab/Turkish individuals as from that region (Veit et al., 2023). In total, 194 AI-generated photos were piloted on their Dutch and Moroccan prototypicality among a representative online sample of 1,477 Dutch ethnic majority members. These photos of faces were selected from the academic dataset created by Generated Photos (*N* = 110) or generated using their AI software (*N* = 84).^[[2]](#footnote-2)^ In total, 74 faces of Moroccan men, 68 Moroccan women, 27 Dutch men, and 25 Dutch women with a neutral expression were tested. Following Ma and colleagues (2021), we aimed for 57 ratings for each photo, and randomly presented a maximum of eight photos to each participant. On average, each photo was rated by 61 participants (*SD* = 2.54, *min =* 53*, max =* 67).

Photos were rated on realness (1 = *yes,* 2 = *no*), attractiveness (1 = *not at all attractive,* 4 = *very attractive*), how prototypical Dutch they looked (1 = *not at all,* 4 = *completely*), and how prototypical Moroccan they looked (1 = *not at all,* 4 = *completely*). First, we selected photos that were rated by at least 80% of participants as real (*N_photos_* = 163). Next, to ensure clear ethnic/national signals, we performed one-sample t-tests against the midpoint of the scales (2.5) to select the faces that were rated significantly higher on prototypicality for one group and significantly lower for the other group. Hence, a Moroccan-looking face in our study was rated significantly higher than 2.5 on the scale of prototypically Moroccan-looking and significantly lower on prototypically Dutch-looking. This resulted in 25 photos of Dutch men, 23 of Dutch women, 25 of Moroccan men, and 10 of Moroccan women. We needed two Dutch men, two Dutch women, five Moroccan men and five Moroccan women for our experiment, and selected those that were perceived as equally attractive and which had the highest prototypicality scores for their respective group. From our final 14 faces, 13 were rated significantly more attractive than the mid-point (2.5) of the scale, and only one photo of a Moroccan woman was not rated significantly different. The selected faces scored on average as follow on prototypicality for their respective group: Dutch men, *M*_Dutchprototypicality_ = 3.09, *SD* = .98, Dutch women, *M*_Dutchprototypicality_ = 2.95, *SD* = .81, Moroccan men, *M*_Moroccanprototypicality_ = 3.05, *SD* = 1.04, and Moroccan women. *M*_Moroccanprototypicality_ = 3.00, *SD* = .94.

# Study 2: Results Pre-test AI-generated Photos

Table S3 shows the descriptive results of our pre-test for the 14 AI-generated photos, per group. It shows the total number of ratings that these faces received, what percentage of participants saw them as real (0/1) and how attractive (1-4) these faces were rated on average. It, moreover, shows how prototypical Dutch (1-4) and Moroccan (1-4) looking these faces were rated and whether these ratings differed significantly from the mid-point of the scales (2.5). All faces shown here were part of our experiment.

*Table S3. Descriptive results of pre-test for AI-generated photos per group (p one-sided).*

|  |  |  |  |  | *Dutch prototypicality* | | | | | *Moroccan prototypicality* | | | | |
| --- | --- | --- | --- | --- | --- | --- | --- | --- | --- | --- | --- | --- | --- | --- |
|  | *N_total_* | *%_real_* | *M_attr_* | SD_attr_ | *M* | *SD* | *t* | *df* | *p* | *M* | *SD* | *t* | *df* | *p* |
| *Dutch men* |  |  |  |  |  |  |  |  |  |  |  |  |  |  |
| 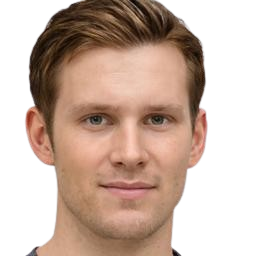 | 63 | 95.24 | 3.19 | .90 | 3.53 | .65 | 12.07 | 58 | <.001 | 1.25 | .71 | -13.49 | 58 | <.001 |
| 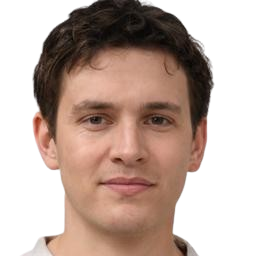 | 64 | 92.22 | 3.00 | 1.07 | 3.36 | .58 | 11.33 | 58 | <.001 | 1.56 | .68 | -10.68 | 58 | <.001 |
|  |  |  |  |  | *Dutch prototypicality* | | | | | *Moroccan prototypicality* | | | | |
|  | *N_total_* | *%_real_* | *M_attr_* | *SD_attr_* | *M* | *SD* | *t* | *df* | *p* | *M* | *SD* | *t* | *df* | *p* |
| *Moroccan men (targets)* |  |  |  |  |  |  |  |  |  |  |  |  |  |  |
| 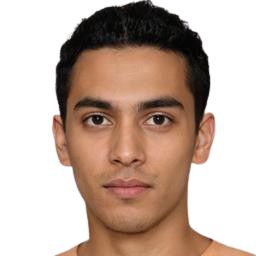 | 59 | 86.44 | 3.18 | .94 | 1.94 | .79 | -4.99 | 49 | <.001 | 2.98 | .71 | 4.75 | 49 | <.001 |
| 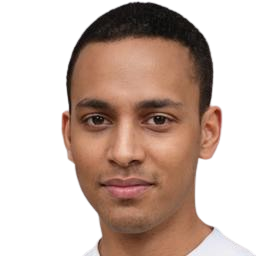 | 62 | 87.10 | 2.85 | 1.04 | 1.85 | .90 | -5.30 | 53 | <.001 | 2.93 | .82 | 3.81 | 53 | <.001 |
| 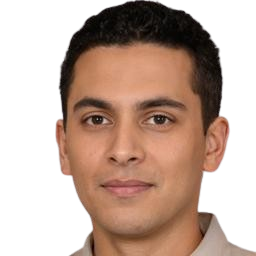 | 58 | 89.66 | 3.02 | 1.06 | 2.12 | .81 | -3.43 | 51 | <.001 | 2.92 | .81 | 3.75 | 51 | <.001 |
|  |  |  |  |  | *Dutch prototypicality* | | | | | *Moroccan prototypicality* | | | | |
|  | *N_total_* | *%_real_* | *M_attr_* | *SD_attr_* | *M* | *SD* | *t* | *df* | *p* | *M* | *SD* | *t* | *df* | *p* |
| *Moroccan men (friends)* |  |  |  |  |  |  |  |  |  |  |  |  |  |  |
| **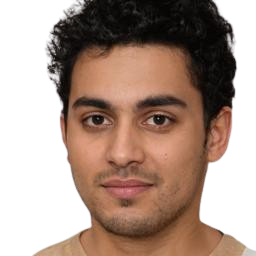** | 60 | 91.7 | 2.91 | 1.22 | 1.82 | .11 | -6.18 | 54 | <.001 | 2.89 | .11 | 3.69 | 54 | <.001 |
| **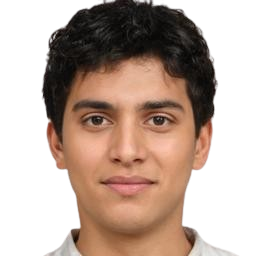** | 63 | 85.7 | 3.30 | .92 | 1.98 | .12 | -4.23 | 53 | <.001 | 2.83 | .11 | 2.99 | 53 | .002 |
| *Dutch women* |  |  |  |  |  |  |  |  |  |  |  |  |  |  |
| 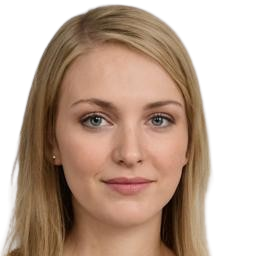 | 58 | 93.10 | 3.00 | .80 | 3.78 | .50 | 18.72 | 53 | <.001 | 1.07 | .43 | -24.49 | 53 | <.001 |
|  |  |  |  |  | *Dutch prototypicality* | | | | | *Moroccan prototypicality* | | | | |
|  | *N_total_* | *%_real_* | *M_attr_* | *SD_attr_* | *M* | *SD* | *t* | *df* | *p* | *M* | *SD* | *t* | *df* | *p* |
| 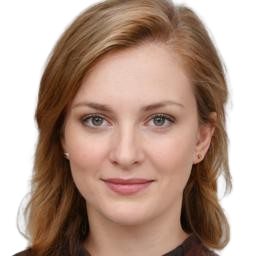 | 65 | 93.85 | 2.90 | .81 | 3.61 | .56 | 15.54 | 60 | <.001 | 1.16 | .61 | -17.09 | 60 | <.001 |
| *Moroccan women (targets)* |  |  |  |  |  |  |  |  |  |  |  |  |  |  |
| 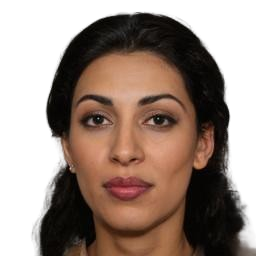 | 56 | 88.89 | 2.95 | 1.09 | 1.75 | .84 | -6.71 | 55 | <.001 | 2.96 | .85 | 4.08 | 55 | <.001 |
| 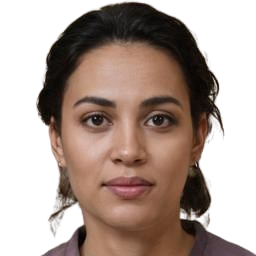 | 60 | 88.3 | 3.08 | 1.05 | 1.92 | .81 | -5.20 | 52 | <.001 | 2.91 | .77 | 3.85 | 52 | <.001 |
|  |  |  |  |  | *Dutch prototypicality* | | | | | *Moroccan prototypicality* | | | | |
|  | *N_total_* | *%_real_* | *M_attr_* | *SD_attr_* | *M* | *SD* | *t* | *df* | *p* | *M* | *SD* | *t* | *df* | *p* |
| 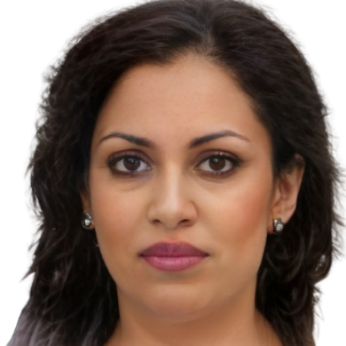 | 61 | 85.2 | 2.37 | .86 | 2.04 | .84 | -3.97 | 51 | <.001 | 2.87 | .69 | 3.84 | 51 | <.001 |
| *Moroccan women (friends)* |  |  |  |  |  |  |  |  |  |  |  |  |  |  |
| 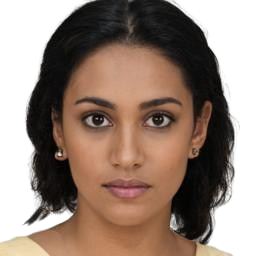 | 62 | 87.1 | 3.41 | .92 | 1.91 | .85 | -5.11 | 53 | <.001 | 2.85 | .86 | 3.02 | 53 | .002 |
| 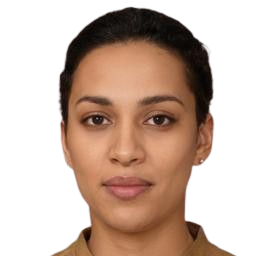 | 62 | 90.3 | 3.18 | .79 | 2.02 | .84 | -4.29 | 55 | <.001 | 2.82 | .77 | 3.14 | 55 | .001 |

# Study 2: Vignettes Friend Group Conditions

In Study 2, ethnic/national background of friends were signalled using AI-generated photos. The introduction was held constant across conditions and resembled the text in Study 1. Below is an example of the four conditions with a female Moroccan-Dutch target. The conditions were the same for men, but with male faces. Text is translated from Dutch.

*Introduction*

On the next page, you will read a description of a fictitious person. We are interested in your impression of her and will ask you questions about her. Please read the text carefully, you cannot return to the text if you go to the next page.

*Introduction of target*

The next questions will be about this person that you see here. She is 30 years old and born and raised in the Netherlands. Her parents were born in Morocco. We are interested in your impression of her and will ask some questions about her.


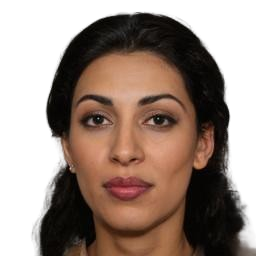


*Condition 1 (Dutch friends)*


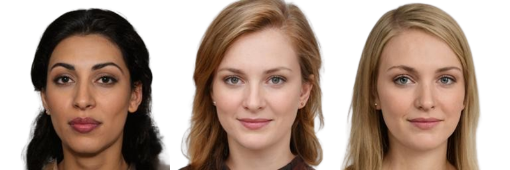


Here you see her together with her two best friends. In her leisure time, she likes to hang out with them. Her friends are very important to her.

*Condition 2 (Moroccan friends)*

*
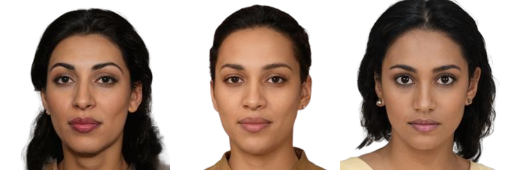
*

Here you see her together with her two best friends. In her leisure time, she likes to hang out with them. Her friends are very important to her.

*Condition 3 (mixed friends)*


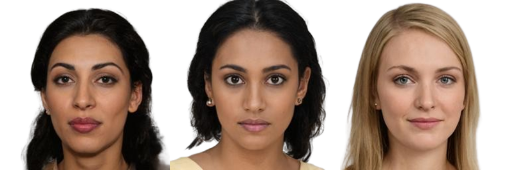


Here you see her together with her two best friends. In her leisure time, she likes to hang out with them. Her friends are very important to her.

*Condition 4 (no friends/control)*

Only introduction and introduction of target were shown.

*Example of a question*


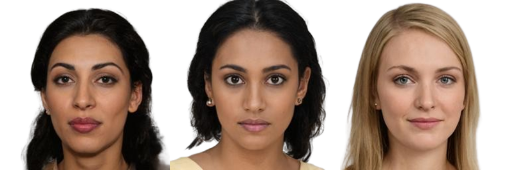


How Dutch is this person in your view?

# Study 1: Ethnic Conception of Nationhood

*Table S4. Correlations of ascription and ethnic conception of nationhood (N = 644).*

|  | 1 | 2 | 3 |
| --- | --- | --- | --- |
| 1. Dutch ascription |  |  |  |
| 1. Dual ascription | .11** |  |  |
| 1. Moroccan ascription | -.49*** | .23*** |  |
| 1. Ethnic conception of nationhood | -.36*** | -.04 | .26*** |

*Note*. ** indicates *p* <.01, *** indicates *p* <.001.

|  | Dutch  ascriptions | | | | Dual  ascriptions | | | Moroccan ascriptions | | |
| --- | --- | --- | --- | --- | --- | --- | --- | --- | --- | --- |
|  | *df* | *F* | *p* | *ɳ^2^p* | *F* | *p* | *ɳ^2^p* | *F* | *p* | *ɳ^2^p* |
| Friend condition | 3, 639 | 37.45 | <.001 | .150 | 3.01 | .030 | .014 | 16.53 | <.001 | .072 |
| Ethnic conception | 1, 639 | 114.22 | <.001 | .152 | .68 | .407 | .001 | 50.87 | <.001 | .074 |

*Table S5. Results of ANCOVA’s for ascriptions by friends condition and ethnic conception of nationhood (N = 644).*

Figure S1 shows the mean dual ascriptions for high (+1 SD) and low (-1 SD) ethnic nationhood endorsers per friend group condition. Post hoc Tukey HSD’s of separate ANOVA’s for the effect of friends group for high and low ethnic nationhood endorses were performed. High ethnic nationhood endorsers ascribed significantly lower levels of dual belonging in the Moroccan friends condition compared to the no friends (control) condition (*Mean diff* = 1.17, *p adj* = .003). On the other hand, low ethnic nationhood endorsers ascribed significantly higher levels of dual belonging in the Moroccan friend condition compared to the Dutch friend condition (*Mean diff* = .77, *p adj* = .023). Further examination of the average Dutch and Moroccan ascriptions for high ethnic nationhood endorsers showed that in the Moroccan friend condition the target was ascribed high levels of Moroccan belonging (*M* = 4.00, *SD* = .97) and low levels of Dutch belonging (*M* = 1.65, *SD =* .75). Which resulted in the lowest dual score compared to the other three friend group conditions. Conversely, low ethnic nationhood endorsers ascribed the target higher levels of Dutch belonging than Moroccan belonging in all conditions except in the Moroccan friend condition where the ascriptions of Dutch (*M =* 2.85, *SD* = 1.03) and Moroccan (*M* = 2.90, *SD* = .93) belonging were fairly similar resulting in a higher dual ascriptions score in the Moroccan friend condition.

*Figure S1. Mean dual ascriptions for high (+1 SD) and low (-1 SD) ethnic nationhood endorsers per friend group condition.*

*
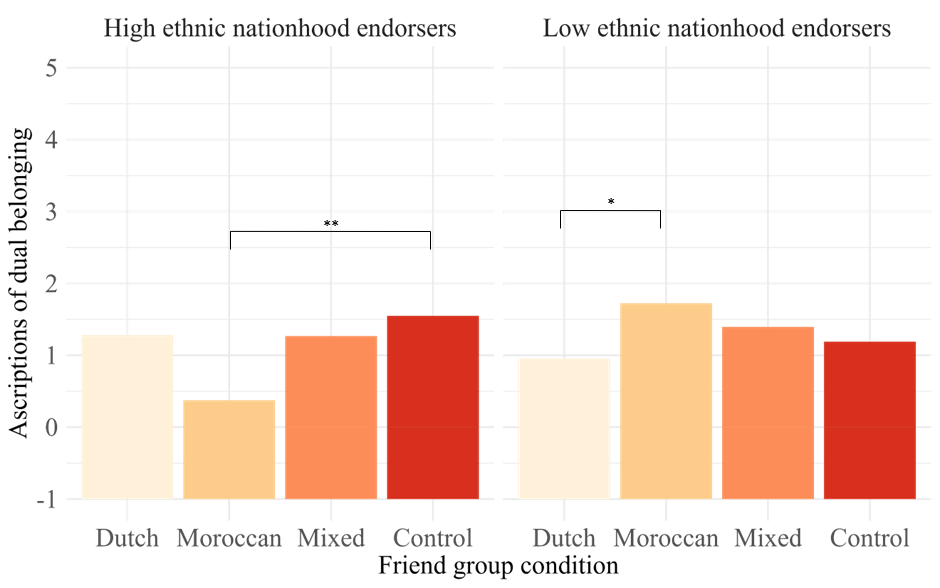
*

*Note.* * indicates significant difference at *p* <.05, ** indicates *p* <.01.

# Study 1: ANCOVA Results of Evaluations

*Table S6. Means and Standard Deviations of stereotypical evaluations in the four conditions (N = 644).*

|  | Friend group conditions | | | | Test of mean differences  (ANOVA) | | |
| --- | --- | --- | --- | --- | --- | --- | --- |
|  | Dutch friends  (*N*  = 168) | Moroccan friends  (*N*  = 144) | Mixed friends  (*N*  = 148) | No friends (control)  (*N*  = 184) | *F* | *p* | *ɳ^2^* |
| Likeability | 3.46_a_  (.74) | 2.89_b_  (.80) | 3.42_a_  (.70) | 3.17_c_  (.78) | 18.13 | <.001 | .078 |
| Trust | 3.39_a_  (.77) | 2.86_b_  (.81) | 3.33_ac_  (.76) | 3.14_c_  (.80) | 13.86 | <.001 | .061 |
| Intelligence | 3.33_a_  (.66) | 2.90_b_  (.64) | 3.30_a_  (.65) | 3.02_b_  (.69) | 15.60 | <.001 | .068 |

*Note.* Standard deviations are presented in parentheses, means with different subscripts differed at *p* < .05 in Tukey’s HSD tests.

*Table S7. Results of separate ANCOVA’s for likeability, trust and intelligence by friends condition and ethnic conception of nationhood (N = 644).*

|  | Likeability | | | | Trust | | |
| --- | --- | --- | --- | --- | --- | --- | --- |
|  | *df* | *F* | *p* | *ɳ_p_ ^2^* | *F* | *p* | *ɳ_p_^2^* |
| Friend condition | 3, 639 | 18.44 | <.001 | .80 | 14.86 | <.001 | .065 |
| Ethnic conception | 1, 639 | 89.02 | <.001 | .122 | 134.85 | <.001 | .174 |

|  | Intelligence | | | |
| --- | --- | --- | --- | --- |
|  | *df* | *F* | *p* | *ɳ_p_^2^* |
| Friend condition | 3, 639 | 14.65 | <.001 | .064 |
| Ethnic conception | 1, 639 | 89.80 | <.001 | .123 |

*Note:* Ethnic nationhood was centred in these analyses.

# Study 1: Results Robustness Check

For the robustness check participants who failed the attention check or manipulation check were included. Table S8, S9 and S10 show the results of the main analyses including all participants.

*Table S8. Means and Standard Deviations of the ascription variables in the four conditions (N = 860).*

|  | Friend group conditions | | | | Test of mean differences  (ANOVA) | | |
| --- | --- | --- | --- | --- | --- | --- | --- |
|  | Dutch friends  (*N*  = 217) | Moroccan friends  (*N*  = 214) | Mixed friends  (*N*  = 215) | No friends (control)  (*N*  = 214) | *F* | *p* | *ɳ^2^* |
| Dutch ascription | 3.51_a_  (1.13) | 2.64_b_  (1.07) | 3.35_a_  (1.13) | 3.27  (1.05) | 26.19 | <.001 | .084 |
| Dual ascription | 1.29  (1.21) | 1.46  (1.33) | 1.55  (1.38) | 1.45  (1.16) | 1.54 | .203 | .005 |
| Moroccan ascriptions | 2.42  (.95) | 3.04  (1.05) | 2.66  (1.04) | 2.60  (.97) | 14.43 | <.001 | .048 |

*Note.* Standard deviations are presented in parentheses, means with different subscripts differed at *p* < .05 in Tukey’s HSD tests.

*Table S9. Results from three ANCOVA’s for the variables Dutch, dual and Moroccan ascriptions with full sample (N = 860).*

|  | Dutch ascriptions | | | | Dual ascriptions | | | | Moroccan ascriptions | | | |
| --- | --- | --- | --- | --- | --- | --- | --- | --- | --- | --- | --- | --- |
|  | *df* | *F* | *p* | *ɳ_p_^2^* | *df* | *F* | *p* | *ɳ_p_^2^* | *df* | *F* | *p* | *ɳ_p_^2^* |
| Friend condition | 3, 852 | 33.26 | <.001 | .104 | 3, 852 | 1.47 | .221 | .005 | 3, 852 | 18.08 | <.001 | .057 |
| Ethnic nationhood | 1 , 852 | 25.58 | <.001 | .141 | 1, 852 | .39 | .531 | .002 | 1, 852 | 3.65 | .056 | .067 |
| Friend condition * Ethnic | 3, 852 | .33 | .802 | .001 | 3, 852 | 5.16 | .002 | .018 | 3, 852 | 2.59 | .052 | .009 |

*Note:* Ethnic nationhood was centred in these analyses.

A MANOVA for likeability, trust and intelligence showed main effects for the friend group condition (*F*(9, 2556) = 6.73, *p* < .001) and ethnic conception of nationhood (*F*(3, 850) = 54.08, *p* < .001), but not for their interaction (*F*(9, 2556) = .70, *p* = .710).

*Table S10. ANCOVA’s results for stereotypical evaluation variables with full sample (N = 860).*

|  |  | Likeability | | | Trust | | | Intelligence | | |
| --- | --- | --- | --- | --- | --- | --- | --- | --- | --- | --- |
|  | *df* | *F* | *p* | *ɳ_p_^2^* | *F* | *p* | *ɳ_p_^2^* | *F* | *p* | *ɳ_p_^2^* |
| Friend condition | 3, 855 | 16.60 | <.001 | .055 | 14.73 | <.001 | .049 | 13.10 | <.001 | .044 |
| Ethnic nationhood | 1, 855 | 111.18 | <.001 | .115 | 154.99 | <.001 | .153 | 115.01 | <.001 | .119 |

*Note:* Ethnic nationhood was centred in these analyses.

# Study 1: Additional Analysis

Participants were asked how close they thought the friend group was (not asked to those in the no friends condition, 1*=* *not close at all* to 5 *= very close*) and to what extent they felt that the target could be a bridge between the Dutch and Moroccan community in the Netherlands (1 = *not at all,* 5 *= definitely*). Table S11 shows the mean, SD and results of the ANOVA’s. There were no significant differences in closeness between the three friends conditions. However, the friend group condition did affect the target's perceived bridging potential. The target was perceived to have less bridging potential in the Moroccan friends condition, compared to the Dutch friends condition (*Mean diff*  = -.63, *p* < .001), mixed friends condition (*Mean diff*  = -.54, *p* < .001), and no friends condition (*Mean diff*  = -.29, *p* = .020). He was also seen as having more bridging potential in the Dutch friends condition compared to the no friends condition (*Mean diff =* .33, *p* = .004).

*Table S11. Means and Standard Deviation for closeness and perceived bridging potential in the four conditions (N = 644).*

|  | Friend group conditions | | | | Test of mean differences  (ANOVA) | | |
| --- | --- | --- | --- | --- | --- | --- | --- |
|  | Dutch friends  (*N*  = 168) | Moroccan friends  (*N*  = 144) | Mixed friends  (*N*  = 148) | No friends (control)  (*N*  = 184) | *F* | *p* | *ɳ^2^* |
| Closeness | 3.77_a_  (.75) | 3.92_a_  (.72) | 3.72_a_  (.75) | N.A. | 2.97 | .052 | .013 |
| Bridge | 3.77_a_  (.87) | 3.15_b_  (1.01) | 3.68_ac_  (.84) | 3.44_c_  (.92) | 14.49 | <.001 | .064 |

*Note.* Standard deviations are presented in parentheses, means with different subscripts differed at *p* < .05 in Tukey’s HSD tests.

# Study 2: Target Differences

Tables S12 to S15 show the results of multiple two-way ANOVA’s for the friend group condition and the three targets for all dependent variables. Results showed that there was a difference between the targets for likeability for male (*F*(6, 423) = 3.23, *p* = .041, *ɳ_p_^2^ =* .015) and female targets (*F*(6, 426) = 5.26, *p* = .006, *ɳ_p_^2^ =* .024). For male targets, target 3 was rated significantly more likable than target 1 (*Mean diff* = .23, *p* = .043). For female targets, target 1 was rated significantly less likeable than target 2 (*Mean diff* = .30, *p* = .005). Additionally, when adding their interaction, there was an interaction effect for female targets between the friend group condition and the targets for Dutch ascriptions (*F*(6, 420) = 3.05, *p* = .006, *ɳ_p_^2^ =* .043). Figure S2 shows that target 1 had a different pattern than target 2 and 3.

*Table S12. Results of two-way ANOVA for all dependent variables for male targets (N = 429).*

|  |  | Dutch ascriptions | | | Dual ascriptions | | | Moroccan ascriptions | | | Likeability | | |
| --- | --- | --- | --- | --- | --- | --- | --- | --- | --- | --- | --- | --- | --- |
|  | *df* | *F* | *p* | *ɳ_p_^2^* | *F* | *p* | *ɳ_p_^2^* | *F* | *p* | *ɳ_p_^2^* | *F* | *p* | *ɳ_p_^2^* |
| Friend condition | 3, 423 | 1.28 | .281 | .009 | .36 | .780 | .003 | 1.69 | .169 | .012 | .21 | .889 | .001 |
| Target | 2, 423 | .56 | .570 | .003 | .34 | .712 | .002 | 1.16 | .314 | .005 | 3.23 | .041 | .015 |

|  |  | Trust | | | Intelligence | | | Attractiveness | | |
| --- | --- | --- | --- | --- | --- | --- | --- | --- | --- | --- |
|  | *df* | *F* | *p* | *ɳ_p_^2^* | *F* | *p* | *ɳ_p_^2^* | *F* | *p* | *ɳ_p_^2^* |
| Friend condition | 3, 423 | 1.03 | .377 | .007 | .17 | .918 | .001 | 2.90 | .035 | .020 |
| Target | 2, 423 | 1.37 | .255 | .006 | 1.46 | .233 | .007 | .39 | .681 | .002 |

*Table S13. Results of two-way ANOVA with interaction term for all dependent variables for male targets (N = 429).*

|  |  | Dutch ascriptions | | | Dual ascriptions | | | Moroccan ascriptions | | | Likeability | | |
| --- | --- | --- | --- | --- | --- | --- | --- | --- | --- | --- | --- | --- | --- |
|  | *df* | *F* | *p* | *ɳ_p_^2^* | *F* | *p* | *ɳ_p_^2^* | *F* | *p* | *ɳ_p_^2^* | *F* | *p* | *ɳ_p_^2^* |
| Friend condition | 3, 417 | 1.07 | .362 | .009 | .54 | .653 | .003 | 1.78 | ,150 | .012 | .57 | .638 | .002 |
| Target | 2, 417 | 1.23 | .294 | .003 | .03 | .970 | .002 | .94 | .392 | .006 | .039 | .962 | .015 |
| Friend condition * target | 6, 417 | .75 | .608 | .011 | .49 | .817 | .007 | 1.33 | .242 | .019 | .43 | .860 | .006 |

|  |  | Trust | | | Intelligence | | | Attractiveness | | |
| --- | --- | --- | --- | --- | --- | --- | --- | --- | --- | --- |
|  | *df* | *F* | *p* | *ɳ_p_^2^* | *F* | *p* | *ɳ_p_^2^* | *F* | *p* | *ɳ_p_^2^* |
| Friend condition | 3, 417 | .96 | .413 | .037 | .05 | .985 | .001 | .968 | .408 | .020 |
| Target | 2, 417 | .00 | .999 | .038 | .32 | .727 | .007 | .444 | .642 | .002 |
| Friend condition * target | 6, 417 | .43 | .858 | .021 | .34 | .914 | .005 | .295 | .939 | .004 |

*Table S14. Results of two-way ANOVA for all dependent variables for female targets (N = 432).*

|  |  | Dutch ascriptions | | | Dual ascriptions | | | Moroccan ascriptions | | | Likeability | | |
| --- | --- | --- | --- | --- | --- | --- | --- | --- | --- | --- | --- | --- | --- |
|  | *df* | *F* | *p* | *ɳ_p_^2^* | *F* | *p* | *ɳ_p_^2^* | *F* | *p* | *ɳ_p_^2^* | *F* | *p* | *ɳ_p_^2^* |
| Friend condition | 3, 426 | 2.50 | .059 | .017 | 1.50 | .213 | .010 | 5.28 | .001 | .036 | 1.61 | .186 | .011 |
| Target | 2, 426 | 2.55 | .079 | .012 | .68 | .506 | .003 | 8.21 | <..001 | .037 | 5.26 | .006 | .024 |

|  |  | Trust | | | Intelligence | | | Attractiveness | | |
| --- | --- | --- | --- | --- | --- | --- | --- | --- | --- | --- |
|  | *df* | *F* | *p* | *ɳ_p_^2^* | *F* | *p* | *ɳ_p_^2^* | *F* | *p* | *ɳ_p_^2^* |
| Friend condition | 3, 426 | .65 | .584 | .005 | 1.72 | .163 | .012 | .63 | .595 | .004 |
| Target | 2, 426 | 2.91 | .055 | .013 | 1.37 | .255 | .006 | 2.63 | .073 | .012 |

*Table S15. Results of two-way ANOVA with interaction term for all dependent variables for female targets (N = 432).*

|  |  | Dutch ascriptions | | | Dual ascriptions | | | Moroccan ascriptions | | | Likeability | | |
| --- | --- | --- | --- | --- | --- | --- | --- | --- | --- | --- | --- | --- | --- |
|  | *df* | *F* | *p* | *ɳ_p_^2^* | *F* | *p* | *ɳ_p_^2^* | *F* | *p* | *ɳ_p_^2^* | *F* | *p* | *ɳ_p_^2^* |
| Friend condition | 3, 420 | 2.09 | .101 | .018 | 1.15 | .329 | .011 | .31 | .816 | .047 | .75 | .524 | .011 |
| Target | 2, 420 | 2.96 | .053 | .012 | .15 | .863 | .003 | 4.19 | .016 | .038 | 1.53 | .217 | .025 |
| Friend condition * target | 6, 420 | 3.05 | .006 | .042 | 1.26 | .276 | .018 | 1.48 | .185 | .021 | 1.55 | .159 | .022 |

|  |  | Trust | | | Intelligence | | | Attractiveness | | |
| --- | --- | --- | --- | --- | --- | --- | --- | --- | --- | --- |
|  | *df* | *F* | *p* | *ɳ_p_^2^* | *F* | *p* | *ɳ_p_^2^* | *F* | *p* | *ɳ_p_^2^* |
| Friend condition | 3, 420 | .57 | .637 | .005 | .24 | .870 | .012 | .52 | .671 | .005 |
| Gender | 2, 420 | 2.80 | .062 | .014 | .49 | .616 | .007 | 2.31 | .101 | .013 |
| Friend condition * target | 6, 420 | 1.51 | .172 | .021 | 1.23 | .288 | .017 | 1.83 | .093 | .025 |

*Note.* ** indicates *p* < .01. * indicates *p* < .05.

*Figure S2*. *Mean Dutch ascription for each female target per friend group condition.*


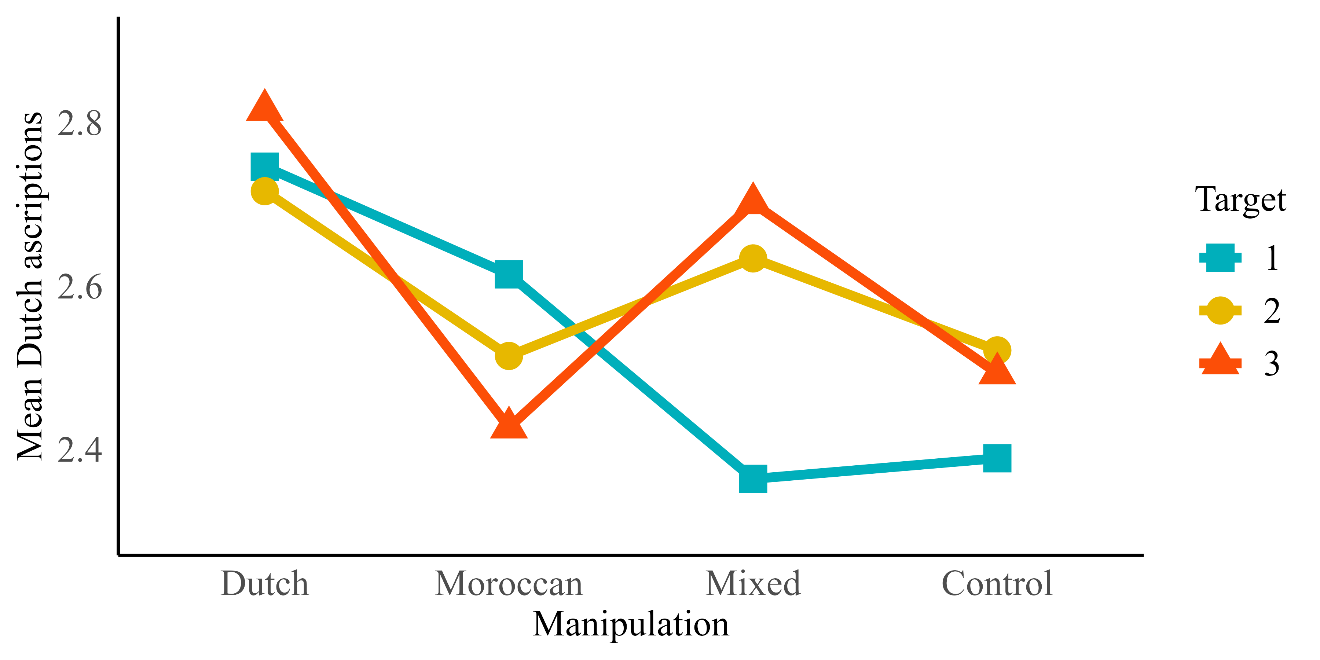


# Study 2: Robustness Without Target 1

Following the above mentioned analyses of target differences, the male and female target 1 were removed and the main analyses for likeability and Dutch ascriptions were repeated as a robustness check. This resulted in a sample of 575 participants, see Table S16 for how these were distributed across the friend conditions. Table S17, S18 and S19 show the results of these analyses. As discussed in the paper, the conclusions remained the same as in the main analyses with all targets included.

*Table S16. Number of participants per friend group condition without target 1 for both male and female targets (N = 575).*

| Male targets | | | | Female targets | | | |
| --- | --- | --- | --- | --- | --- | --- | --- |
| Dutch friends | Moroccan friends | Mixed friends | No friends | Dutch friends | Moroccan friends | Mixed friends | No friends |
| 74 | 73 | 66 | 71 | 76 | 72 | 72 | 71 |

*Table S17. Results of separate ANOVA’s for the friend group condition on Dutch ascriptions and likeability (N = 575).*

|  | Male targets | | | | | | | | Female targets | | | | | | | |
| --- | --- | --- | --- | --- | --- | --- | --- | --- | --- | --- | --- | --- | --- | --- | --- | --- |
|  | Dutch ascriptions | | | | Likeability | | | | Dutch ascriptions | | | | Likeability | | | |
|  | *df* | *F* | *p* | *ɳ^2^* | *df* | *F* | *p* | *ɳ^2^* | *df* | *F* | *p* | *ɳ^2^* | *df* | *F* | *p* | *ɳ^2^* |
| Friend condition | 3, 280 | .55 | .645 | .006 | 3, 280 | .29 | .833 | .003 | 3, 287 | 2.56 | .015 | .036 | 3, 287 | 1.64 | .179 | .017 |

*Table S18. Results of separate ANCOVA’s for the friend group condition and targets’ gender on Dutch ascriptions and likeability (N = 575).*

|  | Dutch ascriptions | | | | | | | | Likeability | | | | | | | |
| --- | --- | --- | --- | --- | --- | --- | --- | --- | --- | --- | --- | --- | --- | --- | --- | --- |
|  | *df* | *F* | *p* | *ɳ_p_^2^* | *df* | *F* | *p* | *ɳ_p_^2^* | *df* | *F* | *p* | *ɳ_p_^2^* | *df* | *F* | *p* | *ɳ_p_^2^* |
| Friend condition | 3, 570 | 2.61 | .051 | .014 | 3, 567 | .58 | .625 | .014 | 3, 570 | .49 | .690 | .003 | 3, 567 | .58 | .625 | .014 |
| Gender | 1, 570 | 3.43 | .064 | .006 | 1, 567 | 2.37 | .124 | .006 | 1, 570 | 1.04 | .309 | .002 | 1, 567 | 2.37 | .124 | .006 |
| Friend condition * Gender | 3, 570 |  |  |  | 3, 567 | 1.24 | .259 | .007 | 3, 570 |  |  |  | 3, 567 | 1.34 | .259 | .007 |

*Table S19. Results of separate ANCOVA’s for friend condition and ethnic conception of nationhood on ascriptions for male (N = 284) and female targets (N = 291).*

|  | Male targets | | | | Female targets | | | | | | |
| --- | --- | --- | --- | --- | --- | --- | --- | --- | --- | --- | --- |
|  | Likeability | | | | Dutch ascriptions | | | | Likeability | | |
|  | *df* | *F* | *p* | *ɳ_p_^2^* | *df* | *F* | *p* | *ɳ_p_^2^* | *F* | *p* | *ɳ_p_^2^* |
| Friend condition | 3, 276 | .16 | .926 | .002 | 3, 283 | 2.18 | .091 | .023 | 1.42 | .237 | .016 |
| Ethnic nationhood | 1, 276 | 15.04 | <.001 | .142 | 1, 283 | .06 | .815 | .143 | 2.91 | .089 | .089 |
| Friend condition *  Ethnic nationhood | 3, 276 | .30 | .825 | .003 | 3, 283 | 6.59 | <.001 | .065 | 1.35 | .258 | .014 |

*Note.* Ethnic nationhood was centred in these analyses.

.

# Study 2: Friend Group Conditions

Table S20 shows the ANOVA results for the effect of friend group condition on each dependent variable for male targets, and Table S21 shows these results for female targets.

*Table S20. Results of separate ANOVA’s for friend group condition on ascriptions for male targets* (*N = 429)*.

|  |  | Dutch ascriptions | | | Dual ascriptions | | | Moroccan ascriptions | | | Likeability | | |
| --- | --- | --- | --- | --- | --- | --- | --- | --- | --- | --- | --- | --- | --- |
|  | *df* | *F* | *p* | *ɳ^2^* | *F* | *p* | *ɳ^2^* | *F* | *p* | *ɳ^2^* | *F* | *p* | *ɳ^2^* |
| Friend condition | 3, 425 | 1.29 | .278 | .009 | .37 | .773 | .003 | 1.65 | .176 | .012 | .23 | .087 | .002 |

|  |  | Trust | | | Intelligence | | | Attractiveness | | |
| --- | --- | --- | --- | --- | --- | --- | --- | --- | --- | --- |
|  | *df* | *F* | *p* | *ɳ^2^* | *F* | *p* | *ɳ^2^* | *F* | *p* | *ɳ^2^* |
| Friend condition | 3, 425 | 1.07 | .362 | .007 | .18 | .909 | .001 | 2.85 | .037 | .020 |

*Table S21. Results of separate ANCOVA’s for friend group condition on ascriptions for female targets (N = 432).*

|  |  | Dutch ascriptions | | | Dual ascriptions | | | Moroccan ascriptions | | | Likeability | | |
| --- | --- | --- | --- | --- | --- | --- | --- | --- | --- | --- | --- | --- | --- |
|  | *df* | *F* | *p* | *ɳ^2^* | *F* | *p* | *ɳ^2^* | *F* | *p* | *ɳ^2^* | *F* | *p* | *ɳ^2^* |
| Friend condition | 3, 428 | 2.68 | .046* | .018 | 1.45 | .228 | .010 | 5.48 | .001 | .037 | 1.79 | .148 | .012 |

|  |  | Trust | | | Intelligence | | | Attractiveness | | |
| --- | --- | --- | --- | --- | --- | --- | --- | --- | --- | --- |
|  | *df* | *F* | *p* | *ɳ^2^* | *F* | *p* | *ɳ^2^* | *F* | *p* | *ɳ^2^* |
| Friend condition | 3, 428 | .76 | .518 | .005 | 1.77 | .152 | .012 | .74 | .527 | .005 |

# Study 2: Descriptive Results Evaluations

*Table S22. Mean and Standard Deviation for stereotypical evaluations per friend group condition.*

|  | Experimental conditions | | | | | | | | | | | | | | | |
| --- | --- | --- | --- | --- | --- | --- | --- | --- | --- | --- | --- | --- | --- | --- | --- | --- |
|  | Men  Dutch friends  (*N*  = 111) | | Men  Moroccan friends  (*N*  = 108) | | Men  Mixed friends  (*N*  = 105) | | Men  No friends (control)  (*N*  = 105) | | Women  Dutch friends  (*N*  = 106) | | Women  Moroccan friends  (*N*  = 107) | | Women  Mixed friends  (*N*  = 110) | | Women  No friends (control)  (*N*  = 109) | |
|  | *M* | *SD* | *M* | *SD* | *M* | *SD* | *M* | *SD* | *M* | *SD* | *M* | *SD* | *M* | *SD* | *M* | *SD* |
| Likeability | 3.00_a_ | .84 | 3.00_a_ | .86 | 2.93_a_ | .83 | 2.93_a_ | .85 | 3.18_a_ | .81 | 2.84_a_ | .79 | 3.01_a_ | .78 | 3.06_a_ | .73 |
| Trust | 3.05_a_ | .85 | 2.96_a_ | .82 | 2.87_a_ | .83 | 2.90_a_ | .85 | 3.25_a_ | .81 | 3.11_a_ | .79 | 3.16_a_ | .78 | 3.10_a_ | .73 |
| Intelligence | 2.98_a_ | .79 | 2.98_a_ | .71 | 2.97_a_ | .63 | 3.04_a_ | .77 | 3.28_a_ | .79 | 3.18_a_ | .72 | 3.25_a_ | .72 | 3.07_a_ | .63 |
| Attractiveness | 2.21_ab_ | 1.02 | 2.30_a_ | 1.02 | 1.92_b_ | .87 | 2.10_ab_ | .99 | 2.99_a_ | 1.06 | 2.81_a_ | 1.05 | 2.81_a_ | 1.03 | 2.83_a_ | 1.06 |
| Closeness | 3.46_a_ | .81 | 3.62_a_ | .86 | 3.45_a_ | .90 | NA | NA | 3.67_a_ | .87 | 3.67_a_ | .79 | 3.46_a_ | .80 | NA | NA |
| Bridging | 3.40_a_ | .97 | 3.34_a_ | .98 | 3.36_a_ | .83 | 3.36_a_ | .94 | 3.62_a_ | .92 | 3.47_a_ | .92 | 3.50_a_ | .84 | 3.39_a_ | .85 |

*Note.* Within men and women, means with different subscripts differed at *p* < .05 in Tukey’s HSD tests.

# Study 2: Effect of Targets’ Gender

Table S23 shows the results of ANCOVA’s with an interaction term for friend group condition and targets’ gender for all dependent variables. The analyses showed no significant interaction effects.

*Table S23. Results of ANCOVA’s with interaction term for friend group condition and gender for all dependent variables (N = 861).*

|  |  | Dutch ascriptions | | | Dual ascriptions | | | Moroccan ascriptions | | | Likeability | | |
| --- | --- | --- | --- | --- | --- | --- | --- | --- | --- | --- | --- | --- | --- |
|  | *df* | *F* | *p* | *ɳ_p_^2^* | *F* | *p* | *ɳ_p_^2^* | *F* | *p* | *ɳ_p_^2^* | *F* | *p* | *ɳ_p_^2^* |
| Friend condition | 3, 853 | 1.31 | .269 | .012 | .35 | .787 | .003 | 1.65 | .176 | .020 | .23 | .873 | .004 |
| Gender | 1, 853 | .49 | .482 | .001 | .64 | .422 | .001 | 2.23 | .136 | <.001 | 2.57 | .109 | .002 |
| Friend condition * Gender | 3, 853 | .64 | .589 | .002 | .89 | .447 | .003 | 1.23 | .298 | .004 | .94 | .421 | .003 |

|  |  | Trust | | | Intelligence | | | Attractiveness | | |
| --- | --- | --- | --- | --- | --- | --- | --- | --- | --- | --- |
|  | *df* | *F* | *p* | *ɳ_p_^2^* | *F* | *p* | *ɳ_p_^2^* | *F* | *p* | *ɳ_p_^2^* |
| Friend condition | 3, 853 | 1.15 | .330 | .005 | .19 | .907 | .002 | 2.65 | .048 | .008 |
| Gender | 1, 853 | 3.02 | .083 | .017 | 9.41 | .002 | .019 | 32.33 | <.001 | .115 |
| Friend condition * Gender | 3, 853 | .32 | .813 | .001 | 1.47 | .222 | .005 | 1.26 | .286 | .004 |

# Study 2: Sample Description

In Study 2, 861 participants remained in the sample. Of these participants 45.53% was male, 54.36% female and 0.12% indicated ‘other’. They were on average 49.78 years old (*SD* = 15.99). Moreover, 17.54% had completed lower, 48.32% medium, and 34.15% higher education. An ANOVA showed that age did not differ significantly between the 24 conditions (*F*(23, 837) = 1.01, *p* = .450) and a chi-squared test showed that there was no significant difference in educational level (*χ^2^*(46) = 27.73, *p* = .985).

# Study 2: Friend Condition and Ethnic Nationhood

*Table S24. Correlations between all variables for male targets (above the diagonal) and female targets (below the diagonal).*

|  | 1 | 2 | 3 | 4 | 5 | 6 | 7 | 8 |
| --- | --- | --- | --- | --- | --- | --- | --- | --- |
| 1. Dutch ascription | - | .34*** | -.41*** | .45*** | .49*** | .42*** | .31*** | -.38*** |
| 1. Dual ascription | .32*** | - | -.18*** | .18*** | .22*** | .16** | .01 | -.23*** |
| 1. Moroccan ascription | -.40*** | -.02 | - | -.32*** | -.37*** | -.33*** | -.36*** | .37*** |
| 1. Likeability | .35*** | .10* | -.26*** | - | .82*** | .67*** | .48*** | -.35*** |
| 1. Trust | .40*** | .10* | -.31*** | .74*** | - | .71*** | .47*** | -.42*** |
| 1. Intelligence | .29*** | .10* | -.19*** | .53*** | .63*** | - | .40*** | -.32*** |
| 1. Attractiveness | .28*** | .05 | -.29*** | .56*** | .55*** | .49*** | - | -.32*** |
| 1. Ethnic conception of nationhood | -.38*** | -.10* | .31*** | -.27*** | -.30*** | -.29*** | -.23*** | - |

Note. *** indicates *p* <.001, ** indicates *p* <.01.

Table S25 shows the results of an ANCOVA for the effect of friend group condition and ethnic conception of nationhood on all dependent variables for male targets, and Table S26 shows the same but including an interaction term. Similarly, Table S27 show the analysis including the interaction term for female targets. For female targets, there was a significant interaction effect on Dutch ascriptions (*F*(3, 424) = 2.97, *p =* .032, *ɳ_p_^2^* = .021). Figure 3 displays the mean levels of Dutch ascriptions for high (+1 SD) and low (-1 SD) on ethnic nationhood endorsers. Post hoc Tukey HSD’s of separate ANOVA’s for the effect of friends group for high and low ethnic nationhood endorses were performed. These showed that high ethnic nationhood endorsers gave less Dutch ascriptions in the mixed friends conditions compared to the Dutch friends condition (*Mean diff* = -.87, *p adj* = .043), but no other contrasts were significant. For low ethnic nationhood endorsers there were no significant differences in ascriptions between the friend group conditions.

*Table S25. Results of ANCOVA’s for friend group condition and ethnic conception of nationhood for male targets* (*N = 429)*.

|  |  | Dutch ascriptions | | | Dual ascriptions | | | Moroccan ascriptions | | | Likeability | | |
| --- | --- | --- | --- | --- | --- | --- | --- | --- | --- | --- | --- | --- | --- |
|  | *df* | *F* | *p* | *ɳ_p_^2^* | *F* | *p* | *ɳ_p_^2^* | *F* | *p* | *ɳ_p_^2^* | *F* | *p* | *ɳ_p_^2^* |
| Friend condition | 3, 424 | 1.72 | .162 | .012 | .61 | .61 | .004 | 1.37 | .250 | .010 | .16 | .921 | .001 |
| Ethnic nationhood | 1, 424 | 73.12 | <.001 | .147 | 25.46 | <.001 | .057 | 68.08 | <.001 | .138 | 59.06 | <.001 | .122 |

|  |  | Trust | | | Intelligence | | | Attractiveness | | |
| --- | --- | --- | --- | --- | --- | --- | --- | --- | --- | --- |
|  | *df* | *F* | *p* | *ɳ_p_^2^* | *F* | *p* | *ɳ_p_^2^* | *F* | *p* | *ɳ_p_^2^* |
| Friend condition | 3, 424 | 1.19 | .315 | .008 | .44 | .725 | .003 | 2.97 | .032 | .021 |
| Ethnic nationhood | 1, 424 | 90.50 | <.001 | .176 | 49.65 | <.001 | .105 | 49.84 | <.001 | .105 |

*Note.* Ethnic nationhood was centred in these analyses.

*Table S26. Results of ANCOVA’s with interaction term for friend group condition and ethnic conception of nationhood for male targets* (*N = 429)*.

|  |  | Dutch ascriptions | | | Dual ascriptions | | | Moroccan ascriptions | | | Likeability | | |
| --- | --- | --- | --- | --- | --- | --- | --- | --- | --- | --- | --- | --- | --- |
|  | *df* | *F* | *p* | *ɳ_p_^2^* | *F* | *p* | *ɳ_p_^2^* | *F* | *p* | *ɳ_p_^2^* | *F* | *p* | *ɳ_p_^2^* |
| Friend condition | 3, 421 | 1.77 | .152 | .012 | .68 | .563 | .004 | 1.38 | .249 | .010 | .15 | .928 | .001 |
| Ethnic nationhood | 1, 421 | 14.26 | <.001 | .147 | .90 | .343 | .057 | 27.32 | <.001 | .139 | 18.80 | <.001 | .123 |
| Friend condition *  Ethnic nationhood | 3, 421 | .46 | .713 | .003 | 1.19 | .314 | .008 | 1.17 | .321 | .008 | .78 | .508 | .006 |

|  |  | Trust | | | Intelligence | | | Attractiveness | | |
| --- | --- | --- | --- | --- | --- | --- | --- | --- | --- | --- |
|  | *df* | *F* | *p* | *ɳ_p_^2^* | *F* | *p* | *ɳ_p_^2^* | *F* | *p* | *ɳ_p_^2^* |
| Friend condition | 3, 421 | 1.17 | .321 | .008 | .52 | .667 | .003 | 2.92 | .034 | .021 |
| Ethnic nationhood | 1, 421 | 20.51 | <.001 | .176 | 11.34 | .001 | .106 | 10.75 | .001 | .107 |
| Friend condition *  Ethnic nationhood | 3, 421 | .41 | .744 | .003 | 1.55 | 201 | .011 | 2.13 | .095 | .015 |

*Note.* Ethnic nationhood was centred in these analyses.

*Table S27. Results of ANCOVA’s with interaction term for friend group condition and ethnic conception of nationhood for female targets (N = 432).*

|  |  | Dutch ascriptions | | | Dual ascriptions | | | Moroccan ascriptions | | | Likeability | | |
| --- | --- | --- | --- | --- | --- | --- | --- | --- | --- | --- | --- | --- | --- |
|  | *df* | *F* | *p* | *ɳ_p_^2^* | *F* | *p* | *ɳ_p_^2^* | *F* | *p* | *ɳ_p_^2^* | *F* | *p* | *ɳ_p_^2^* |
| Friend condition | 3, 424 | 2.15 | .093 | .013 | 1.72 | .163 | .013 | 4.18 | .006 | .029 | 2.29 | .078 | .016 |
| Ethnic nationhood | 1, 424 | 3.17 | .076 | .140 | .57 | .451 | .012 | 2.27 | .133 | .086 | 2.56 | .111 | .075 |
| Friend condition *  Ethnic nationhood | 3, 424 | 2.97 | .032 | .021 | .37 | .772 | .003 | 1.09 | .353 | .008 | 1.50 | .214 | .010 |

|  |  | Trust | | | Intelligence | | | Attractiveness | | |
| --- | --- | --- | --- | --- | --- | --- | --- | --- | --- | --- |
|  | *df* | *F* | *p* | *ɳ_p_^2^* | *F* | *p* | *ɳ_p_^2^* | *F* | *p* | *ɳ_p_^2^* |
| Friend condition | 3, 424 | .59 | .620 | .004 | 1.03 | .381 | .007 | .87 | .455 | .006 |
| Ethnic nationhood | 1, 424 | 8.90 | .003 | .089 | 9.37 | .002 | .082 | 2.39 | .123 | .053 |
| Friend condition *  Ethnic nationhood | 3, 424 | .25 | .863 | .002 | .57 | .636 | .004 | .48 | .698 | .003 |

*Note.* Ethnic nationhood was centred in these analyses.

*Figure S3. Mean Dutch ascriptions for female targets for high (+1 SD) and low (-1 SD) ethnic nationhood endorsers per friend group condition.*


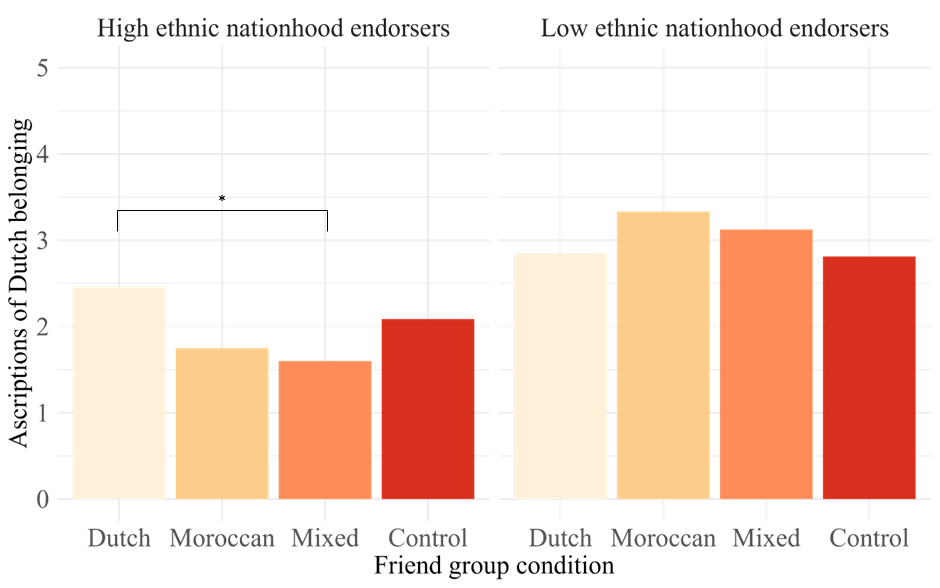


*Note.* * indicates significant difference at *p* <.05.

# Study 2: Results Robustness Checks

Table S28 to S32 show the results of the main analyses including the participants who failed the attention check (*N* = 933).

*Table S28. Results of ANOVA’s for friend group condition for male targets for all dependent variables full sample* (*N = 459)*.

|  |  | Dutch ascriptions | | | Dual ascriptions | | | Moroccan ascriptions | | | Likeability | | |
| --- | --- | --- | --- | --- | --- | --- | --- | --- | --- | --- | --- | --- | --- |
|  | *df* | *F* | *p* | *ɳ^2^* | *F* | *p* | *ɳ^2^* | *F* | *p* | *ɳ^2^* | *F* | *p* | *ɳ^2^* |
| Friend condition | 3, 455 | 1.16 | .326 | .008 | .16 | .923 | .001 | 2.30 | .077 | .015 | .38 | .765 | .003 |

|  |  | Trust | | | Intelligence | | | Attractiveness | | |
| --- | --- | --- | --- | --- | --- | --- | --- | --- | --- | --- |
|  | *df* | *F* | *p* | *ɳ^2^* | *F* | *p* | *ɳ^2^* | *F* | *p* | *ɳ^2^* |
| Friend condition | 3, 455 | 1.59 | .190 | .010 | .30 | .824 | .002 | 2.76 | .042 | .018 |

*Table S29. Results of ANOVA’s for friend group condition for female targets for all dependent variables full sample (N = 474).*

|  |  | Dutch ascriptions | | | Dual ascriptions | | | Moroccan ascriptions | | | Likeability | | |
| --- | --- | --- | --- | --- | --- | --- | --- | --- | --- | --- | --- | --- | --- |
|  | *df* | *F* | *p* | *ɳ^2^* | *F* | *p* | *ɳ^2^* | *F* | *p* | *ɳ^2^* | *F* | *p* | *ɳ^2^* |
| Friend condition | 3, 470 | 3.75 | .011 | .023 | 1.40 | .243 | .009 | 5.73 | .001 | .035 | 2.39 | .068 | .015 |

|  |  | Trust | | | Intelligence | | | Attractiveness | | |
| --- | --- | --- | --- | --- | --- | --- | --- | --- | --- | --- |
|  | *df* | *F* | *p* | *ɳ^2^* | *F* | *p* | *ɳ^2^* | *F* | *p* | *ɳ^2^* |
| Friend condition | 3, 470 | 1.14 | .332 | .007 | 2.45 | .063 | .015 | 1.17 | .321 | .007 |

*Table S30. Results of ANCOVA’s for the friend group condition and targets’ gender for all dependent variables full sample (N = 933).*

|  |  | Dutch ascriptions | | | Dual ascriptions | | | Moroccan ascriptions | | | Likeability | | |
| --- | --- | --- | --- | --- | --- | --- | --- | --- | --- | --- | --- | --- | --- |
|  | *df* | *F* | *p* | *ɳ_p_^2^* | *F* | *p* | *ɳ_p_^2^* | *F* | *p* | *ɳ_p_^2^* | *F* | *p* | *ɳ_p_^2^* |
| Friend condition | 3, 925 | 1.16 | .325 | .013 | .15 | .928 | .003 | 2.28 | .078 | .022 | .39 | .762 | .005 |
| Gender | 1, 925 | 1.57 | .211 | .002 | .82 | .365 | .000 | 2.05 | .153 | .001 | 3.18 | .075 | .003 |
| Friend condition * Gender | 3, 925 | .71 | .548 | .002 | .82 | .482 | .003 | 1.20 | .309 | .004 | 1.26 | .288 | .004 |

|  |  | Trust | | | Intelligence | | | Attractiveness | | |
| --- | --- | --- | --- | --- | --- | --- | --- | --- | --- | --- |
|  | *df* | *F* | *p* | *ɳ_p_^2^* | *F* | *p* | *ɳ_p_^2^* | *F* | *p* | *ɳ_p_^2^* |
| Friend condition | 3, 925 | 1.71 | .162 | .006 | .30 | .824 | .003 | 2.59 | .052 | .008 |
| Gender | 1, 925 | 3.51 | .061 | .019 | 9.01 | .003 | .019 | 36.69 | <.001 | .117 |
| Friend condition * Gender | 3, 925 | .85 | .469 | .003 | 1.74 | .158 | .006 | 1.42 | .236 | .005 |

*Table S31. Results of ANCOVA’s for the friend group condition and ethnic conception of nationhood for all dependent variables for male targets full sample (N = 459).*

|  |  | Dutch ascriptions | | | Dual ascriptions | | | Moroccan ascriptions | | | Likeability | | |
| --- | --- | --- | --- | --- | --- | --- | --- | --- | --- | --- | --- | --- | --- |
|  | *df* | *F* | *p* | *ɳ_p_^2^* | *F* | *p* | *ɳ_p_^2^* | *F* | *p* | *ɳ_p_^2^* | *F* | *p* | *ɳ_p_^2^* |
| Friend condition | 3, 451 | 1.33 | .263 | .008 | .29 | .830 | .002 | 2.12 | .097 | .014 | .36 | .784 | .002 |
| Ethnic nationhood | 1, 451 | 12.82 | <.001 | .131 | .73 | .394 | .052 | 29.10 | <.001 | .136 | 20.04 | <.001 | .123 |
| Friend condition *  Ethnic nationhood | 3, 451 | .69 | .556 | .005 | 1.11 | .344 | .007 | 1.10 | .350 | .007 | 1.17 | .320 | .008 |

|  |  | Trust | | | Intelligence | | | Attractiveness | | |
| --- | --- | --- | --- | --- | --- | --- | --- | --- | --- | --- |
|  | *df* | *F* | *p* | *ɳ_p_^2^* | *F* | *p* | *ɳ_p_^2^* | *F* | *p* | *ɳ_p_^2^* |
| Friend condition | 3, 451 | 1.70 | .165 | .011 | .58 | .632 | .003 | 2.71 | .044 | .018 |
| Ethnic nationhood | 1, 451 | 21.69 | <.001 | .174 | 13.29 | <.001 | .108 | 10.62 | .001 | .101 |
| Friend condition *  Ethnic nationhood | 3, 451 | .46 | .708 | .003 | 1.32 | .267 | .009 | 2.08 | .102 | .014 |

*Note.* Ethnic nationhood was centred in these analyses.

*Table S32. Results of ANCOVA’s for the friend group condition and ethnic conception of nationhood for all dependent variables for female targets full sample (N = 474).*

|  |  | Dutch ascriptions | | | Dual ascriptions | | | Moroccan ascriptions | | | Likeability | | |
| --- | --- | --- | --- | --- | --- | --- | --- | --- | --- | --- | --- | --- | --- |
|  | *df* | *F* | *p* | *ɳ_p_^2^* | *F* | *p* | *ɳ_p_^2^* | *F* | *p* | *ɳ_p_^2^* | *F* | *p* | *ɳ_p_^2^* |
| Friend condition | 3, 466 | 3.55 | .014 | .021 | 1.49 | .216 | .010 | 4.86 | .002 | .031 | 2.66 | .047 | .017 |
| Ethnic nationhood | 1, 466 | 4.91 | .027 | .126 | .05 | .822 | .007 | 3.82 | .051 | .091 | 4.67 | .031 | .061 |
| Friend condition *  Ethnic nationhood | 3, 466 | 3.05 | .028 | .019 | .89 | .445 | .006 | .91 | .437 | .006 | 1.36 | .253 | .009 |

|  |  | Trust | | | Intelligence | | | Attractiveness | | |
| --- | --- | --- | --- | --- | --- | --- | --- | --- | --- | --- |
|  | *df* | *F* | *p* | *ɳ_p_^2^* | *F* | *p* | *ɳ_p_^2^* | *F* | *p* | *ɳ_p_^2^* |
| Friend condition | 3, 466 | 1.08 | .358 | .007 | 2.05 | .107 | .012 | 1.19 | .314=5 | .008 |
| Ethnic nationhood | 1, 466 | 12.48 | <.001 | .080 | 11.69 | <.001 | .070 | 3.99 | .046 | .453 |
| Friend condition *  Ethnic nationhood | 3, 466 | .64 | .589 | .004 | 1.31 | .270 | .008 | .30 | .825 | .002 |

*Note.* Ethnic nationhood was centred in these analyses.

# Study 2: Additional Analyses

Similar to Study 1, we also examined the closeness of the friend group and the bridging potential of the target (See Table S22 above). On average, the male and female friend group were rated quite similar on closeness. Moreover, separate ANOVA’s for male (*F*(2, 321) = 1.37, *p* = .255, *ɳ^2^* = .008) and female (*F*(2, 320) = 2.34, *p* = .098, *ɳ^2^* = .014) targets showed that closeness did not differ based on the friend group condition. Additionally, both male and female targets were also rated similarly on their bridging potential, and there were no significant differences between the friend groups for male targets (*F*(3, 424) = .73, *p* = .975, *ɳ^2^* = .001) or female targets (*F*(1, 428) = 1.33, *p* = .264, *ɳ^2^* = .009).

# Deviation of Final Study to Stage 1 or OSF Preregistration

A first version of the introduction and methods of this paper was accepted as a registered report. Any deviations of the final manuscript to the Stage 1 submission or the preregistration on OSF (<https://doi.org/10.17605/OSF.IO/T6FBR>) are described here.

Our final study differs on several points from the Stage 1 submission. First, based on reviewers’ comments to our Stage 1 submission, we changed our sample size in the preregistration and consequently the final study (p. 9/18 of the paper). In the Stage 1 paper, we calculated our sample size using G*Power. However, as our designs included an interaction we were advised to use the InteractionPoweR Shiny app, an app for calculating a-priori sample size for linear regression analyses including interactions (Finsaas et al., 2021). Therefore the required sample sizes to detect a medium main effect size with 90% power increased from 231 to 612 participants (Study 1), and 300 to 665 participants (Study 2).

Second, in our Stage 1 submission we had only included ascriptions of national belonging and ascriptions of dual belonging as dependent variables, and ethnic conception of nationhood as independent variable. Following comments from our reviewers, we decided to also include variables that measured the evaluation of the targets: perceived likeability, intelligence and trustworthiness (Based on the stereotype content model, Cuddy et al., 2008), closeness of the friend group and the bridging potential of the target. This allowed us to explore downstream consequence of ascriptions.

Third, the design of Study 2 changed slightly between our Stage 1 submission and the pre-registration/final submission. Our initial design was a 2 (male/female) x 4 (friend group) design in which participants would have rated three friend groups and all individuals. However, a group of experts remarked that rating multiple targets and their friends could elicit reactivity. To avoid this, we opted for a 2 (male/female) x 4 (friend groups) x 3 (targets) design, in which participants only saw one friend group and only rated the target. Accordingly, our analyses also changed to fit this design. In addition, to make Study 2 more similar to Study 1, we also included an introduction of the target similar to Study 1 (see p. 14 of supplementary material).

Finally, although our pre-registration and final paper were almost identical, there was one minor deviation in the measurement of ethnic conception of nationhood (p. 12/19 of the paper). We deviated from our preregistration, in both Study 1 and 2, because we incorrectly stated that three items would be used to measure ethnic conception of nationhood instead of two. We decided to not include the third item ‘a real Dutchmen is attached to the traditional Dutch lifestyle’ because this theoretically captures a cultural conception of nationhood (Reijerse et al., 2013). As a robustness check we conducted the analyses including the third item, which did not change the main conclusions.

1. In our pilot study, 71.0% remained after removing those who did not finish the questionnaire, did not pass the attention check, or had failed one or two manipulation checks. Hence, to ensure enough power, we aimed for a greater total sample than our a-priori analysis revealed. [↑](#footnote-ref-1)
2. Generated Photos <https://generated.photos/datasets> [↑](#footnote-ref-2)
